# Physiological response to fetal intravenous lipid emulsion

**DOI:** 10.1042/CS20231419

**Published:** 2024-02-02

**Authors:** Brian D. Piccolo, Athena Chen, Samantha Louey, Kent L.R. Thornburg, Sonnet S. Jonker

**Affiliations:** 1 USDA/ARS-Arkansas Children’s Nutrition Center, Little Rock, AR, U.S.A.; 2 Department of Pediatrics, University of Arkansas for Medical Sciences, Little Rock, AR, U.S.A.; 3 Department of Pathology, Oregon Health and Science University, Portland, OR, U.S.A.; 4 Center for Developmental Health, Knight Cardiovascular Institute, Oregon Health and Science University, Portland, OR, U.S.A.

**Keywords:** fetus, lipid metabolism, lipidomics, parenteral nutrition, preterm birth

## Abstract

In preterm neonates unable to obtain sufficient oral nutrition, intravenous lipid emulsion is life-saving. The contribution of post-conceptional level of maturation to pathology that some neonates experience is difficult to untangle from the global pathophysiology of premature birth. In the present study, we determined fetal physiological responses to intravenous lipid emulsion. Fetal sheep were given intravenous Intralipid 20® (*n* = 4 females, 7 males) or Lactated Ringer’s Solution (*n* = 7 females, 4 males) between 125 ± 1 and 133 ± 1 d of gestation (term = 147 d). Manufacturer’s recommendation for premature human infants was followed: 0.5–1 g/kg/d initial rate, increased by 0.5–1 to 3 g/kg/d. Hemodynamic parameters and arterial blood chemistry were measured, and organs were studied postmortem. Red blood cell lipidomics were analyzed by LC-MS. Intravenous Intralipid did not alter hemodynamic or most blood parameters. Compared with controls, Intralipid infusion increased final day plasma protein (*P*=0.004; 3.5 ± 0.3 vs. 3.9 ± 0.2 g/dL), albumin (*P* = 0.031; 2.2 ± 0.1 vs. 2.4 ± 0.2 g/dL), and bilirubin (*P*<0.001; conjugated: 0.2 ± 0.1 vs. 0.6 ± 0.2 mg/dL; unconjugated: 0.2 ± 0.1 vs. 1.1 ± 0.4 mg/dL). Circulating IGF-1 decreased following Intralipid infusion (*P*<0.001; 66 ± 24 vs. 46 ± 24 ng/mL). Compared with control Oil Red O liver stains (median score 0), Intralipid-infused fetuses scored 108 (*P*=0.0009). Lipidomic analysis revealed uptake and processing of infused lipids into red blood cells, increasing abundance of saturated fatty acids. The near-term fetal sheep tolerates intravenous lipid emulsion well, although lipid accumulates in the liver. Increased levels of unconjugated bilirubin may reflect increased red blood cell turnover or impaired placental clearance. Whether Intralipid is less well tolerated earlier in gestation remains to be determined.

## Introduction

Preterm newborns face a steep challenge to thrive given their immature physiological systems. Adding to the stress that preterm neonates face is the developmentally early introduction of high circulating lipid levels due to oral or intravenous lipid emulsion nutrition for therapeutic purposes [1]. Although it is necessary to provide high lipid levels for brain development in preterm neonates [2,3], the capacity of the immature physiological system to handle exogenous lipids is not well understood.

Preterm neonates that receive lipid emulsion parenteral nutrition (PN) sometimes develop morbidities, including fat accumulation in the lung [4], cardiac pathologies [5,6], and PN-associated liver disease (PNALD) [7–9]. A clear understanding of the pathophysiological response to high circulating lipids in the preterm newborn is complicated by the other simultaneous physiological challenges of preterm birth. It has been convincingly argued that preterm infants should not be expected to follow developmental timelines derived from fetuses of the same postconceptional age [10]. Nevertheless, studying the response of the preterm fetus to lipid emulsion PN may help elucidate the particular physiological responses to high lipid exposure, without the other physiological challenges required for survival in the clinical setting.

In the present study, we infused Intralipid at clinically relevant doses into fetal sheep at 85–90% of gestation to determine how elevated lipids affect fetal hemodynamic stability, blood chemistry, and indicators of metabolic regulation, and how the fetus disposes of administered lipids, including which at-risk organs take up excess lipids. Further, we characterized lipid composition profiles post-infusion using LC-MS-based lipidomics profile of red blood cells (RBCs), as RBC fatty acid composition is a biomarker of nutritional and metabolic conditions affecting cellular function throughout the body [11]. All physiological changes can be attributed to PN lipid, as we isolated this experimental variable from the other physiological impacts associated with preterm birth by studying fetuses.

## Methods

### Animals

All animal experiments were approved by the Institutional Animal Care and Use Committee (#IP0007) and conducted at Oregon Health & Science University, which is accredited by AAALAC International. Healthy ewes with good body conditions carrying timed-bred twin fetuses were obtained from a local supplier. At 119 ± 1 days of gestational age (dGA), ewes underwent sterile surgery to place fetal arterial and venous catheters, as previously described [12], except without the use of atropine. Ewes received subcutaneous Buprenex (0.3 mg buprenorphine HCl; Covetrus, OH, U.S.A.) and sustained release buprenorphine (0.05 mg kg^−1^, ZooPharm, CO, U.S.A.) immediately following surgery. Surgical recovery was 6 ± 1 d. Exclusion criteria were fetal congenital abnormalities, death, or deviations from the flock historical 90% confidence interval for fetal body mass, arterial pH, hematocrit or arterial partial pressure of oxygen (PO_2_). Twelve ewes were allocated to the study; one ewe was excluded from analysis due to profound fetal anemia on day 0.

### Fetal monitoring and experimental model

Acclimatized ewes were housed in metabolic crates with free access to food and water, and the ability to stand or lie down at will [13]. This arrangement allowed continuous fetal hemodynamic monitoring and infusion. Catheter patency was maintained by continuous very low-volume infusion of heparinized Lactated Ringer’s Solution (Minipuls 3, Gilson, Middleton, WI, U.S.A.). Hemodynamic variables were extracted from the record prior to the morning feeding and cleaning. Catheters were connected to a bridge amplifier and recorder (PowerLab, ADInstruments, Colorado Springs, CO, U.S.A.) via in-line transducers (Transpac, Abbott, Abbott Park, IL, U.S.A.) corrected for transducer voltage drift and normalized to intra-amniotic pressure. Heart rate was determined from arterial waveform. An arterial blood sample was run on a Radiometer ABL 825 (Radiometer America, Cleveland, OH) for pH, arterial partial pressure of carbon dioxide (PCO_2_), PO_2_, total hemoglobin, oxygen (O_2_) content, oxy-hemoglobin (O2-Hb) saturation, glucose, and lactate. Hematocrit was assessed by microcapillary centrifugation, and plasma protein by refractometry. On days 0, 4, and 8, plasma (collected with heparin and EDTA anticoagulation) and red blood cell (RBC) samples were separated for later lipid analysis. Packed RBCs were washed twice in normal saline, and then lysed in an equal volume of filtered water. Serum, plasma, and lysed RBCs were frozen at −80°C.

Following assessment of fetal hemodynamics and arterial blood sampling, infusions were initiated or adjusted. Experimental fetuses were selected from each twin pair at random, the other was assigned to the control group. The control group included by chance 7 females and 4 males, while the experimental group included 4 females and 7 males. Experimental fetuses received an infusion of Intralipid 20® according to the manufacturer’s recommendations for premature human infants [4], which is an initial dose of 0.5–1 g kg^−1^ d^−1^, increased by 0.5–1 g kg^−1^ d^−1^ to a maximum of 3 g kg^−1^ d^−1^. At the time of experiment, infusion was based entirely on the predicted fetal weight [14]. Following necropsy, we back-calculated fetal weight to determine actual dose achieved: our initial infusion rate (day 0) was 0.7 ± 0.1 g kg^−1^ d^−1^ (0.142 ± 0.03 ml kg^−1^ h^−1^), at day 4 the rate reached 2.6 ± 0.5 g kg^−1^ d^−1^, (0.540 ± 0.10 ml kg^−1^ h^−1^), and on the final day the rate was 2.8 ± 0.5 g kg^−1^ d^−1^ (0.595 ± 0.11 ml kg^−1^ h^−1^, daily rate values missing for 1 subject). Control fetuses received Lactated Ringer’s Solution at an equal volume to the Intralipid infusion rate of their twin. Gestational age on day 0 was 125 ± 1 dGA (comparable to approximately 33 weeks post conception in humans) and at the conclusion of the experiment (day 8) was 133 ± 1 dGA.

At the conclusion of the experiment, ewes were humanely euthanized with an intravenous overdose of a commercial sodium pentobarbital solution. Fetuses received a bolus dose of 10 ml heparin and 10 ml saturated KCl via the umbilical vein to arrest the heart in diastole. Fetal weight and sex were recorded, and then the heart was dissected. A small left ventricular midventricular section was removed for cryopreservation in Tissue-Tek OCT (Sakura), as were sections of the right rostral lung lobe, the rostral portion of the left liver lobe, and a B-type placentome.

### Assessment of plasma lipids

Plasma lipid analysis was performed at the Lipoprotein Analytical Core of OHSU’s Knight Cardiovascular Institute with masked group identifications. For quantification of total plasma cholesterol, triglycerides and phospholipid concentrations, EDTA-anticoagulated plasma samples were analyzed in duplicate using Cholesterol Liquid Reagents (Pointe Scientific, Canton, MI), Triglycerides Liquid Reagents (Pointe Scientific) and Phospholipids C assay (Fujifilm Wako Diagnostics, Mountain View, CA), respectively. For cholesterol, absorbance was measured at 490 nm; for triglycerides, absorbance was measured at 540 nm; and for phospholipids, absorbance was measured at 600 nm using a microplate reader (SpectraMax iD3, Molecular Devices, San Jose, CA). Cholesterol and glycerol standards (Pointe Scientific) and phospholipid standards (Fujifilm Wako Diagnostics) were used to determine their plasma concentrations. Inter-assay coefficients for cholesterol, triglycerides and phospholipids were 0.68, 2.95, and 2.21, respectively. Intra-assay coefficients for cholesterol, triglycerides and phospholipids were 1.89, 6.33 and 11.03, respectively. Plasma lipid data were log transformed from mg dL^−1^ for analysis.

### RBC lipidomics

RBC lipidomic measurements were made at the Oregon State University Linus Pauling Institute with masked group identifications. RBC samples (40 µl/each) were mixed with 460 µl of cold methylene chloride:isopropanol:methanol (25:10:65, v:v:v), and with 0.01% butylated hydroxytoluene, by vortex at maximum speed for 30 s. The mixture was incubated at −20°C for 1 h, then centrifuged at 13,000 rpm for 10 min at 4°C. Supernatant (98 µl), SPLASH® Lipidomix® internal standards (2 µl) from Avanti Lipids (Alabaster, AL, U.S.A.) and pooled quality control samples were transferred to LC-MS vials and immediately vortexed at maximum speed for 5 s. Prepared samples were stored at −80°C until analysis. LC-MS analysis was performed using a Shimadzu UPLC system (Columbia, MD) coupled with a quadrupole TOF mass spectrometer system (AB SCIEX, TripleTOF 5600) in negative mode. Separation was performed on a Waters ACQUITY CSH C18 column (1.7 µm, 2.1 × 100 mm). Mobile phase A consisted of acetonitrile:water (60:40) with 10 mM ammonium acetate and mobile phase B consisted of isopropanol:acetonitrile (90:10) with 10 mM ammonium acetate. LC-MS parameters were described previously [15]. Identification of lipids was achieved using PeakView Software Version 1.2 (AB SCIEX) based on accurate masses and retention times of each lipid and the lipid library introduced by Cajka et al [16]. After annotation, MultiQuant Software version 3.0.2 (AB SCIEX) was applied to quantify lipids.

### Fetal circulating hormones

Fetal hormones were measured from EDTA-anticoagulated plasma samples by the Endocrine Technologies Core of the Oregon National Primate Research Center. All assays were validated for use in sheep EDTA plasma prior to assaying samples. Values less than the limit of detection were reported as half-way between the limit of detection and zero. Insulin was measured using a bovine ELISA (Alpco, Salem, NH); intra-assay coefficient of variance was 4.3%, inter-assay coefficient of variance was 6.9%; eight samples were below the 0.25 ng ml^−1^ limit of detection. Insulin-like growth factor 1 (IGF-1) was measured using a human ELISA (R&D Systems, Minneapolis, MN). Intra-assay coefficient of variance was 0.7%; inter-assay coefficient of variance was 7.2%; one sample was below the 6.25 ng ml^−1^ limit of detection. Insulin-like growth factor 2 (IGF-2) was measured using human ELISA (R&D Systems). Intra-assay coefficient of variance was 2.3%; inter-assay coefficient of variance was 4.4%. Norepinephrine was measured using the 2-CAT ELISA (LDN, Nordhorn, Germany). Intra-assay coefficient of variance was 9.1%; inter-assay coefficient of variance was 16.9%. There were no values were below the limit of detection for IGF-2 or norepinephrine.

### Assessment of fetal liver function

A fetal liver panel was measured from the terminal day frozen heparin-anticoagulated plasma by the clinical Core Lab at Oregon Health & Science University. Low albumin levels are indicative of liver dysfunction [17]. Alkaline phosphatase and aspartate aminotransferase are biochemical markers of liver cell damage [18]. Bilirubin is a pigment produced by the breakdown of RBCs processed in the liver. Unconjugated (indirect) bilirubin is calculated from total bilirubin minus conjugated (direct) bilirubin. Conjugated bilirubin has been processed by the liver [19]. All assays were run on the Beckman DxC 700 according to the manufacturer specifications for each product insert (Beckman Coulter, Brea, CA, U.S.A.).

### Oil red O staining

OCT-embedded tissues were sectioned and stained with Oil Red O and scored according to criteria developed and validated to diagnose milk aspiration in pulmonary macrophages from pediatric bronchoalveolar lavage samples [20]. In brief, slides were coded to obscure their treatment groups, 100 parenchymal cells from representative areas of each slide were scored by a pathologist (AC) according to their Oil Red O opacity, the scores were summed within a tissue for each animal.

### Statistics

Significance was defined at *α* = 0.05 except where *α* = 0.025 to correct for multiple comparisons (noted in text).

Dichotomous variables were assessed by Fisher’s exact test. Oil Red O scores were first visually assessed to determine similarity of response to treatment by sex, and then were analyzed by Mann–Whitney. These analyses were carried out in GraphPad Prism (v.10.1.0).

Normality of continuous data was assessed by Shapiro–Wilk’s test (*P*>0.05). Homogeneity of variances was assessed by Levene’s test for equality of variances. Outliers were assessed as being 1.5 box-lengths or more from the edge of the box in a boxplot. Fetal weights and liver panel parameters were assessed by two-way ANOVA (by sex and treatment). Hemodynamic and arterial chemistry parameters were assessed by mixed three-way ANOVA (by day, sex and treatment) with the Greenhouse-Geisser correction for sphericity. Main effects were not considered if the interaction term was significant. Multiple comparisons with the Bonferroni correction were performed if indicated. These statistical analyses were carried out in SPSS (v.29.0.0.0).

For lipidomic analysis, normalization methods were compared to reduce mean/median relative standard deviation of pooled quality control samples and to increase stability in data distribution across samples. The systematic error removal using random forest (SERRF) normalization procedure was used [21]. Principal component analysis and metabolite distribution plots identified no samples as outliers. Metabolites were assessed for univariate outliers using an iterative Grubbs test; 16 total outliers were identified and removed, representing ∼0.1% of all data. Data removed from univariate outlier assessment were imputed using the weighted K-nearest neighbors (KNN) imputation method [22]. Visualization of metabolomics data include Principal Component Analysis (PCA) and heatmaps. Data were scaled to unit variance prior to PCA and heatmap visualization. Hierarchical clustering of Euclidian distance was used to order samples and metabolites in heatmaps. Differential abundance analysis of lipid species was performed using linear mixed models in a 2 × 3 design with experimental groups and time points as main effects and individual fetus as a random effect. All main effects and interaction terms are adjusted for multiple comparisons using the Benjamini and Hochberg false discovery rate correction [23].

## Results

### Circulating fetal plasma lipids

**Figure 1:**
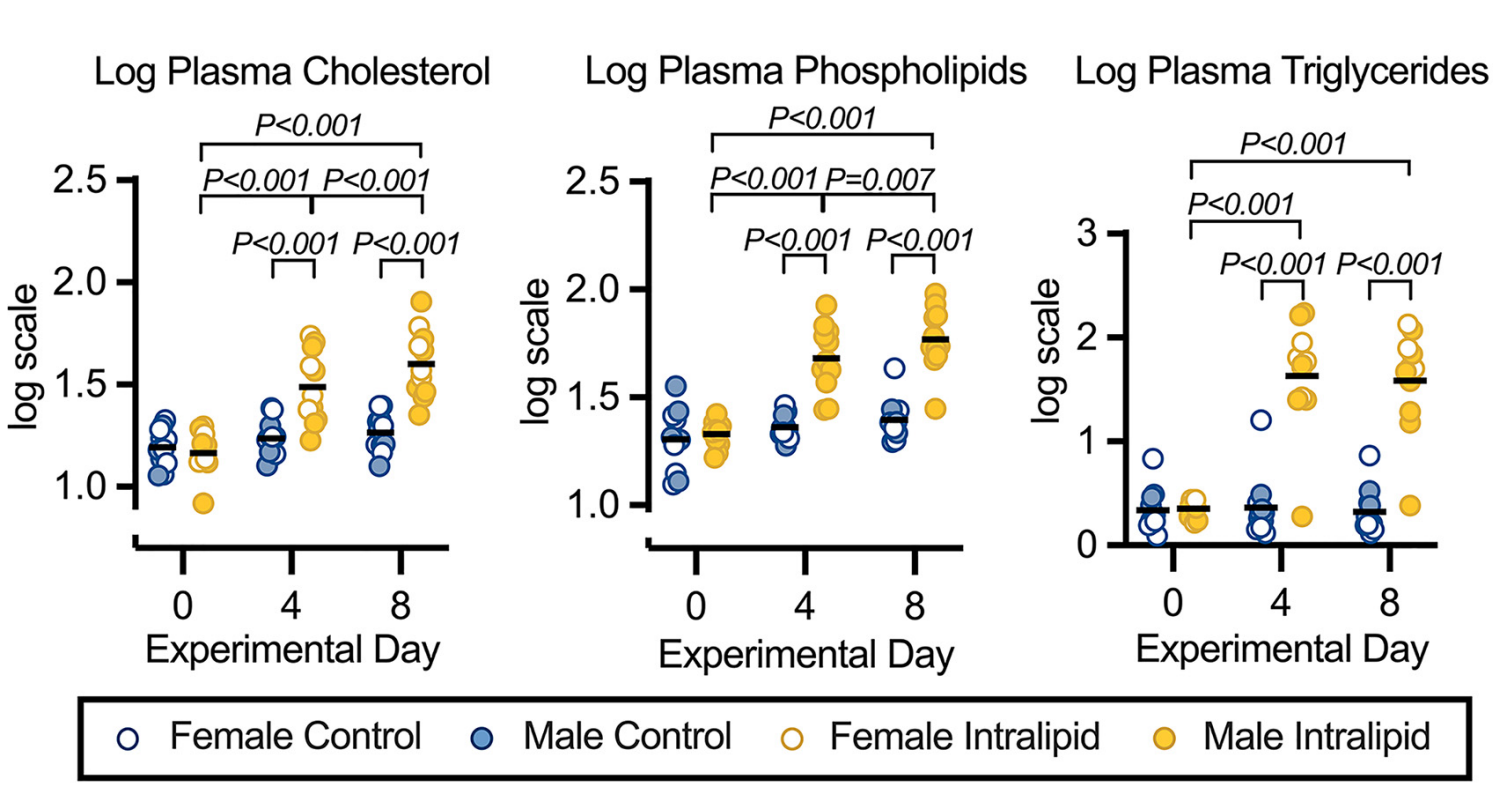
Increased plasma lipids during intravenous Intralipid Fetal plasma cholesterol, phospholipid, and triglyceride levels in fetuses receiving intravenous Intralipid or Lactated Ringer’s Solution for 8 days. Number for Control female = 7, male = 4; Intralipid female = 4, male = 7. Individual data points shown with bar representing mean (sexes combined). Mixed measures three-way ANOVA (within factors by Day, and between factors by treatment and sex) with the Greenhouse-Geisser correction for sphericity. If indicated, multiple comparisons made with Bonferroni correction. For complete statistical analysis, see Table 1.

Circulating fetal plasma lipids are shown in Figure 1 and Supplementary Table S1, and statistical analysis is provided in Tables 1 and Supplementary Table S2. Plasma lipids on experimental day 0 were low and similar between the groups (cholesterol: 1.179 ± 0.019; phospholipids: 1.317 ± 0.017; triglycerides: 0.345 ± 0.0105; all values log transformed from mg dL^−1^). Intralipid infusion increased plasma lipids at day 4 (vs. day 0 and vs. Control *P*<0.001 for all lipid species). Cholesterol concentrations continued to increase to day 8 (vs. days 0 and 4 and vs. Control, each *P*<0.001). Phospholipid concentrations also continued to increase to day 8 (vs. day 0 and vs. Control, each *P*<0.001; vs. day 4, *P*=0.004). Triglyceride concentrations were not further increased at day 8 (vs. day 0 and vs. Control, each *P*<0.001).

**Table 1:** *P*-values from statistical analysis of hemodynamic and arterial blood chemistry parameters

	Three-way interaction	Two-way interactions			Main effects			Subgroup characteristics		
		*Test only if three-way interaction is NS*			*Test only if two- and three-way interactions are NS*			Normal distribution	Homogeneity of variances	Outliers
	Treatment × Day × Sex	Treatment × Day	Day × Sex	Treatment × Sex	Day	Treatment	Sex			
**Arterial pressure** (mmHg)	0.557	0.797	**0.043 *i***	0.290	–	–	–	8/8	2/2	2 *l*
**Venous pressure** (mmHg)	1.000	0.834	0.319	0.630	0.147	0.823	0.293	7/8 *j*	1/2 *k*	1 *l*
**Heart rate** (bpm)	0.058	0.328	0.374	0.982	**<0.001**	0.777	0.056	7/8 *j*	2/2	1 *l*
**pH**	0.712	0.754	0.947	0.267	0.769	0.357	0.758	7/8 *j*	2/2	0
**Hematocrit** (%)	0.970	0.114	0.311	0.383	0.400	0.869	0.908	8/8	2/2	1 *l*
**Total hemoglobin** (g dL^−1^)	0.892	0.292	0.803	0.264	0.346	0.768	0.670	8/8	2/2	0
**PCO_2_** (mmHg)	0.440	0.722	0.445	0.153	0.203	0.913	0.443	8/8	2/2	1 *l*
**PO_2_** (mmHg)	0.616	0.416	0.525	0.299	**0.002**	0.231	0.966	7/8 *j*	2/2	0
**O_2_-Hb saturation** (%)	0.303	0.199	0.434	0.378	0.611	0.129	0.415	8/8	2/2	1 *l*
**O_2_ content** (ml dl^−1^)	0.584	0.168	0.513	0.161	0.519	0.158	0.543	8/8	1/2 *k*	0
**Plasma protein** (g dl^−1^)	0.244	**0.003** *b*	0.726	0.803	–	–	–	8/8	2/2	2 *l*
**Glucose** (mmol l^−1^)	**0.046** *a*	–	–	–	–	–	–	6/8 *j*	2/2	2 *l*
**Lactate** (mmol l^−1^)	0.577	0.623	0.893	0.419	0.114	0.503	0.284	8/8	2/2	1 *l*
**Insulin** (ng ml^−1^)	0.613	**0.028** *c*	0.399	0.921	–	–	–	7/8 *j*	2/2	1 *l*
**IGF-1** (ng ml^−1^)	0.080	**0.004** *d*	0.321	0.708	–	–	–	8/8	2/2	5 *l*
**IGF-2** (ng ml^−1^)	0.855	0.242	0.907	0.456	**0.033**	0.802	0.438	7/8 *j*	2/2	4 *l*
**Norepinepherine** (pg ml^−1^)	0.565	**0.037** *e*	0.891	0.888	–	–	–	8/8	0/2 *k*	1 *l*
**Log cholesterol, plasma**	0.349	**<0.001** *f*	0.721	0.489	–	–	–	12/12	1/3 *k*	0
**Log phospholipids, plasma**	0.141	**<0.001** *g*	0.922	0.492	–	–	–	12/12	2/3 *k*	1 *l*
**Log triglycerides, plasma**	0.457	**<0.001** *h*	0.493	0.144	–	–	–	11/12 *j*	1/3 *k*	1 *l*

Number for Control females = 7, males = 4; Intralipid females = 4, males = 7; except number for pH Control females = 3, males = 3; Intralipid females = 2, males = 4. Mixed measures three-way ANOVA (three levels of repeated measures for plasma lipids, 2 levels for all other variables) with the Greenhouse-Geisser correction for sphericity. If indicated, multiple comparisons made with Bonferroni correction. Not significantly different (NS). Hemoglobin (Hb).

(a) *P*-values for simple two-way interaction following significant three-way interaction using Bonferroni correction for multiple comparisons and adjusting to family-wise *α* = 0.025: Treatment × Day Females = 0.207, Males = 0.784.

*P*-values for significant simple main effects following significant Treatment × Day interaction using Bonferroni correction for multiple comparisons and adjusting to family-wise *α* = 0.025:

(b) Day 0 vs. Day 8 Control = **0.002**, Intralipid **<0.001**; Control vs. Intralipid Day 8 = **0.004**.

(c) Control Day 0 vs. Day 8 = **0.018**.

(d) Intralipid Day 0 vs. Day 8 **<0.001**.

(e) No significant differences (Day 8 Control vs. Intralipid = 0.042).

(f) Intralipid Day 0 vs. Day 4 **<0.001**, Day 0 vs. Day 8 **<0.001**, Day 4 vs. Day 8 **<0.001**; Intralipid vs. Control Day 4 **<0.001**, Day 8 **<0.001**.

(g) Intralipid Day 0 vs. Day 4 **<0.001**, Day 0 vs. Day 8 **<0.001**, Day 4 vs. Day 8 **= 0.007**; Intralipid vs. Control Day 4 **<0.001**, Day 8 **<0.001**.

(h) Intralipid Day 0 vs. Day 4 **<0.001**, Day 0 vs. Day 8 **<0.001**; Intralipid vs. Control Day 4 **<0.001**, Day 8 **<0.001**.

(i) Simple main effects following significant Day × Sex interaction using Bonferroni correction for multiple comparisons: Day 0 vs Day 8 Female = 0.578, Male = **0.022**.

(j) Only indicated proportion of subgroups were normally distributed. Although ANOVAs are fairly robust to deviations from normality, interpret results with caution.

(k) Only indicated proportion of subgroups had homogeneity of variances. Although ANOVAs are fairly robust to heterogeneity of variance, interpret results with caution.

(l) Number of outliers found and determined to be biologically relevant and included in analysis.

### Fetal hemodynamics and arterial blood chemistry

**Figure 2:**
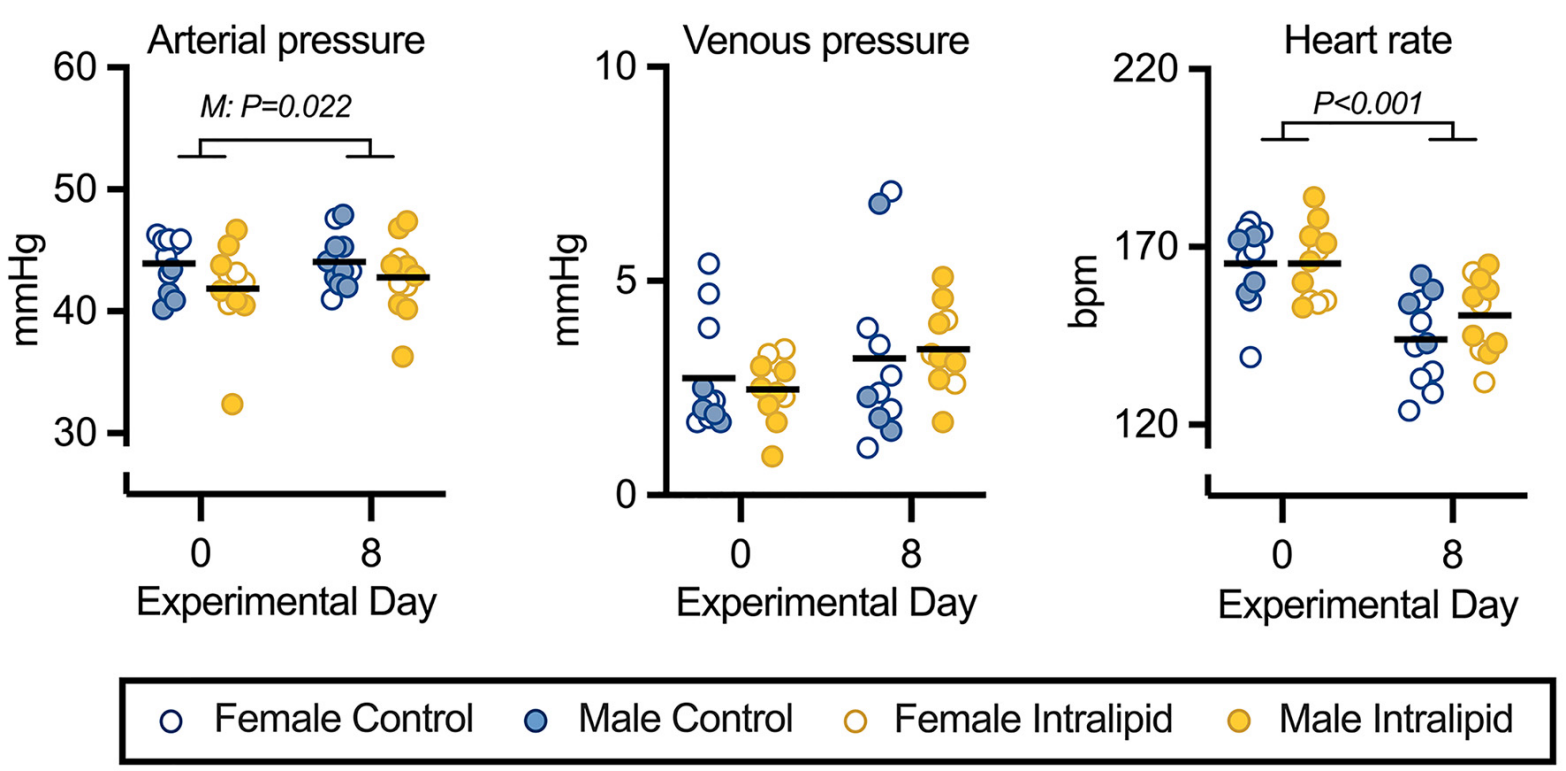
Fetal hemodynamic parameters following intravenous Intralipid Fetal central arterial pressure, central venous pressure, and heart rate in fetuses receiving intravenous Intralipid or lactated Ringer’s solution. Number for control females = 7, males (M) = 4; Intralipid females = 4, males = 7. Individual data points shown with bar representing mean (sexes combined). Mixed measures three-way ANOVA (within factors by Day, and between factors by treatment and sex) with the Greenhouse-Geisser correction for sphericity. If indicated, multiple comparisons made with Bonferroni correction. For complete statistical analysis, see Table 1.

Fetal hemodynamic parameters shown in Figure 2 and Supplementary Table S3, and statistical analysis is provided in Tables 1 and Supplementary Table S2. Independent of treatment, arterial pressure increased 1.5 mmHg between days 0 and 8 in male fetuses (*P*=0.022) but did not change in female fetuses. Venous pressures did not differ between conditions. Heart rate declined between days 0 and 8, independent of treatment or sex, as is typical with maturation (*P*<0.001) [13,24].

**Figure 3:**
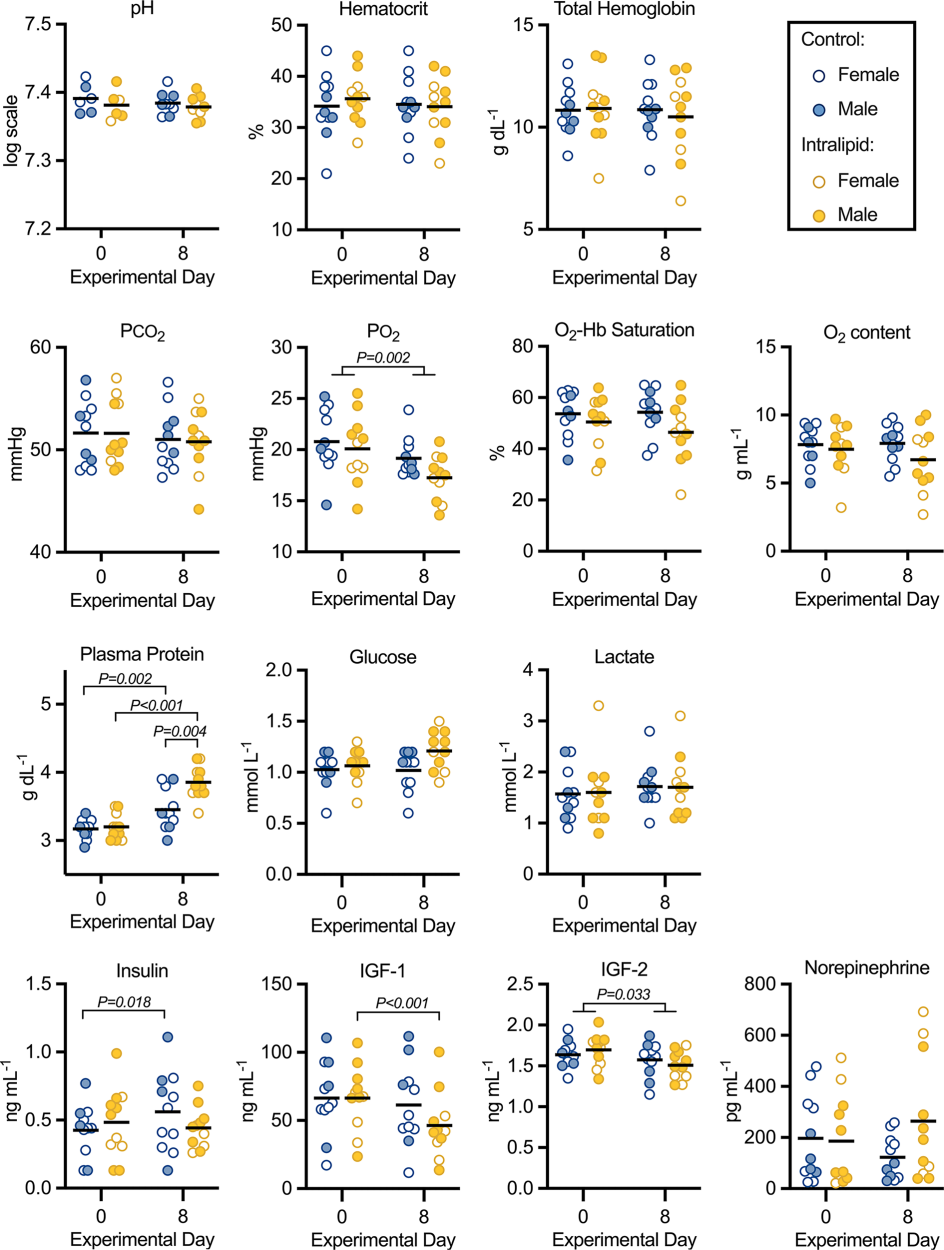
Arterial blood gas and chemistry parameters following intravenous Intralipid Fetal arterial pH, plasma protein, hematocrit, total hemoglobin, partial pressure of CO_2_ (PCO_2_), partial pressure of O_2_ (PO_2_), O_2_-hemoglobin (Hb) saturation, O_2_ content, plasma protein, glucose, lactate, plasma insulin, plasma IGF-1, plasma IGF-2 and plasma norepinepherine in fetuses receiving intravenous Intralipid or lactated Ringer’s solution. Number for control females = 7, males = 4; Intralipid females = 4, males = 7; except for pH control females = 3, males = 3; Intralipid females = 2, males = 4. Individual data points shown with bar representing mean (sexes combined). Mixed measures three-way ANOVA (within factors by day, and between factors by treatment and sex) with the Greenhouse-Geisser correction for sphericity. If indicated, multiple comparisons made with Bonferroni correction. For complete statistical analysis, see Table 1.

Fetal arterial blood chemistry parameters shown in Figure 3 and Supplementary Table S3, and statistical analysis is provided in Tables 1 and Supplementary Table S2. Arterial hematocrit, pH, partial pressure of carbon dioxide (PCO_2_), total hemoglobin concentration, oxyhemoglobin saturation, oxygen content, and lactate levels were unchanged by experimental day, treatment, or sex. PO_2_ was reduced slightly with advancing age irrespective of sex or treatment (*P*=0.002). Advancing gestational age also increased plasma protein in both treatment groups (*P*=0.0164) and, at day 8, plasma protein was greater in the Intralipid group than in controls (*P*=0.004, α = 0.025). Although for blood glucose there was a significant three-way interaction among treatment, day and sex (*P*=0.046), simple two-way interactions were not significantly different.

### Fetal growth

**Figure 4:**
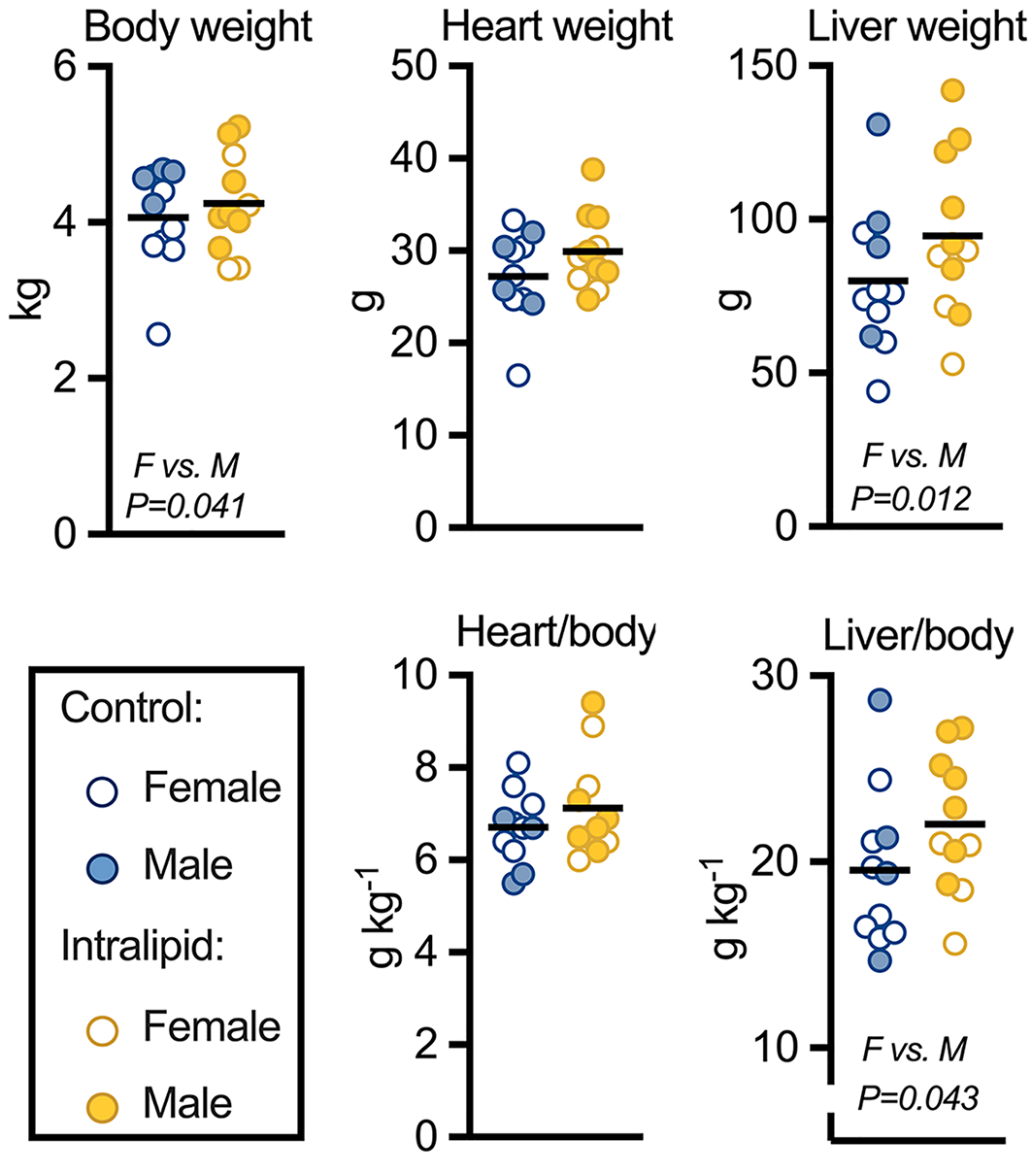
Body and organ weights following Intralipid infusion Fetal body, heart, and liver weights were measured following intravenous Intralipid or lactated Ringer’s solution for 8 days. Number for control females (F) = 7, males (M) = 4; Intralipid females = 4, males = 7. Individual data points shown with bar representing mean (sexes combined). Two-way ANOVA (between factors by treatment and sex). For complete statistical analysis, see Table 2.

Fetal body and organ weights are shown in Figure 4 and Table 2 and Supplementary Table S4. Eight days of Intralipid infusion at clinically relevant rates did not affect terminal body weight. Females, however, were 13% lighter than males irrespective of treatment (*P*=0.041). Likewise, liver weight was unaffected by treatment, but female livers were 29% lighter (*P*=0.012). The weight ratio of liver to body followed a similar pattern, being 18% less in females than in males (*P*=0.043). In contrast, neither treatment nor sex affected heart weight or the weight ratio of heart to body.

**Table 2:** *P*-values from statistical analysis of fetal weights and liver function panel

	Two-way interaction	Main effects		Subgroup data characteristics		
		*Test only if two-way interaction is NS*		Normal distribution	Homogeneity of variances	Outliers
	Treatment × Sex	Treatment	Sex			
**Body weight** (kg)	0.556	0.938	**0.041**	4/4	Yes	1^c^
**Heart weight** (g)	0.727	0.308	0.303	4/4	Yes	1^c^
**Liver weight** (g)	0.801	0.468	**0.012**	4/4	Yes	2^c^
**Heart/body** (g kg^−1^)	0.455	0.211	0.275	3/4^a^	Yes	1^c^
**Liver/body** (g kg^−1^)	0.467	0.365	**0.043**	4/4	Yes	0
**Albumin** (g dL^−1^)	0.094	**0.031**	0.381	4/4	Yes	1^c^
**Alkaline phosphatase** (U L^−1^)	0.772	0.693	0.62	4/4	Yes	1^c^
**Aspartate aminotransferase** (U L^−1^)	0.466	0.343	0.536	4/4	Yes	1^c^
**Bilirubin, unconjugated** (mg dL^−1^)	0.422	**<0.001**	0.159	2/4^a^	No^b^	0
**Bilirubin, conjugated** (mg dL^−1^)	0.915	**<0.001**	0.672	2/4^a^	Yes	2^c^
**Globulin** (g dL^−1^)	0.707	0.213	0.542	4/4	Yes	1^c^

Number for control females = 7, males = 4; Intralipid females = 4, males = 7. Two-way ANOVA. Not significantly different (NS).

a Only indicated number of subgroups were normally distributed. Although ANOVAs are fairly robust to deviations from normality, interpret results with caution.

b Although ANOVAs are fairly robust to heterogeneity of variance, interpret results with caution.

c Outliers determined to be biologically relevant and included in analysis.

At necropsy, no fetuses in the control group were stained with meconium, while 4/11 of the experimental fetuses were stained (*P*=0.0902).

### Fetal RBC lipids

**Figure 5:**
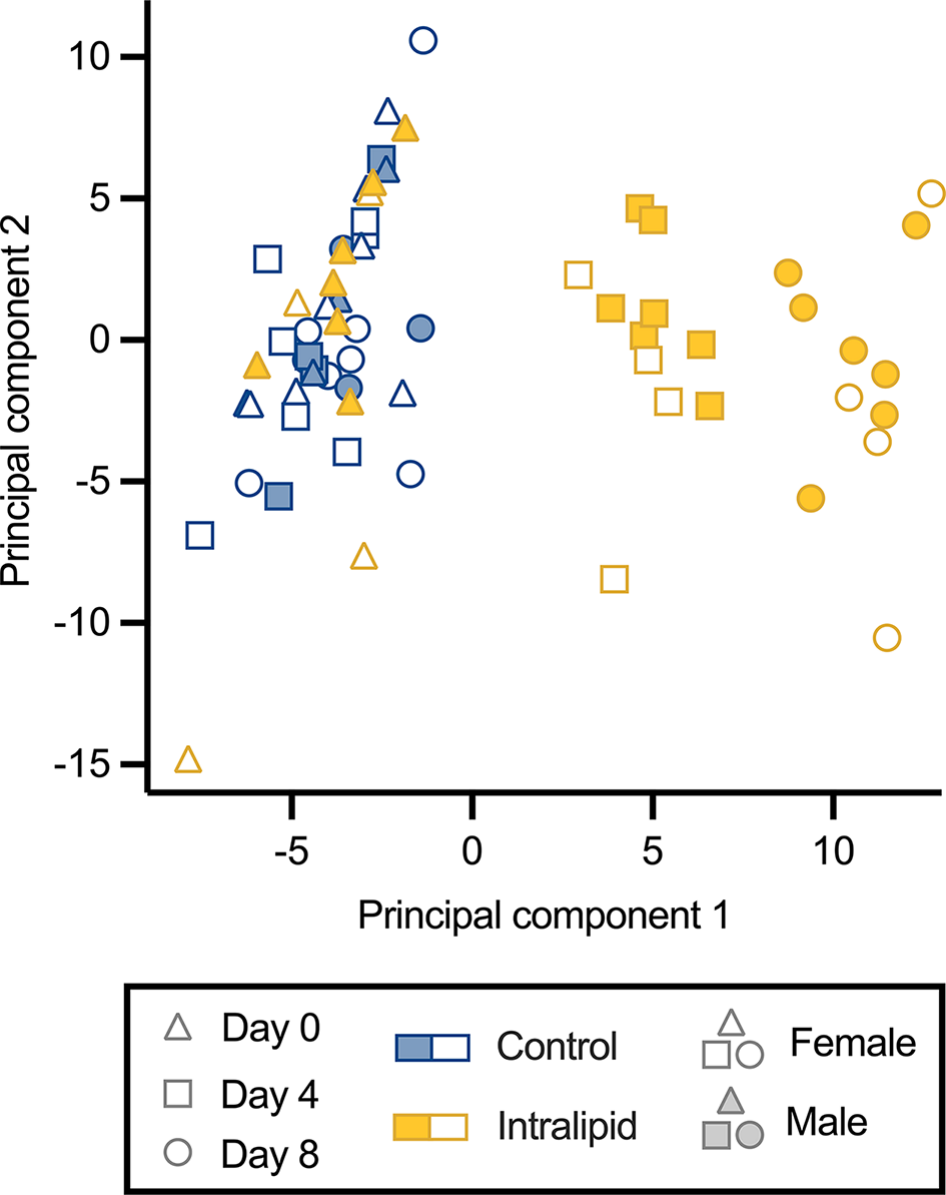
Principal component analysis of fetal RBC lipids during Intralipid infusion Relative concentrations of lipids within fetal RBCs were determined by LC-MS following intravenous Intralipid (*n*=11) or lactated Ringer’s solution (*n*=11) infusion for 8 days. Unsupervised principal component analysis clearly clusters control fetuses (days 0, 4, 8) and day 0 of Intralipid fetuses. Within the Intralipid group, day 4 is separate from day 8. There is no clear clustering by sex.

Visualization of RBC lipidomics data by principal component analysis (a statistical method to summarize information in order to reveal strong patterns) indicated a robust separation of day 4 and day 8 fetuses along component 1 (Figure 5; 21% of explained variance), suggesting that the Intralipid infusion altered the lipid characteristics of fetal RBCs. In contrast with the fetuses receiving the Intralipid infusion, fetuses receiving the lactated Ringer’s solution on days 4 and 8 clustered with day 0 animals, suggesting no differentiation of lipid RBC profiles due to gestational progression. Intra-individual variation was noted in control fetuses using an undirected hierarchical clustering analysis, whereas fetuses receiving the Intralipid solution generally separated by days 4 and 8 (Supplementary Figure S1).

**Figure 6:**
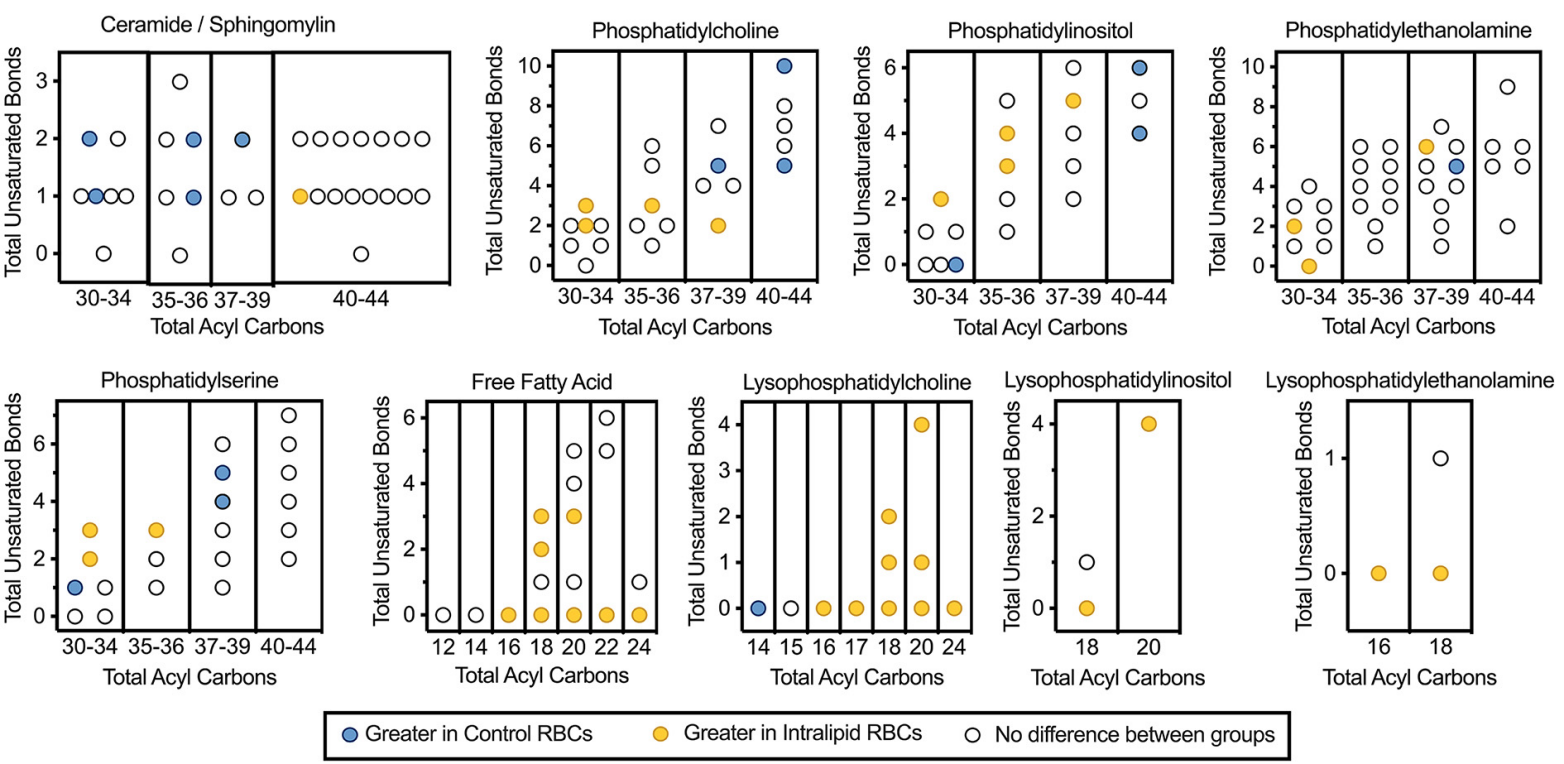
Lipid profiles of fetal RBCs following Intralipid infusion Relative concentrations of lipids within fetal RBCs were determined by LC-MS. Following intravenous Intralipid (*n*=11), 37 of the 163 detected lipid species were elevated, and 15 were depressed compared with controls (*n*=11). Univariate analysis was performed in a linear mixed model 2 × 3 approach (experimental groups by time points) with individual fetus as a random effect (data from days 0 and 4 are in the supplement). All main effects and interaction terms are adjusted for multiple comparisons using the Benjamini and Hochberg false discovery rate correction.

Significant interactions distinguishing Intralipid and control groups across time by total carbons and unsaturated bonds are presented for days 0, 4 (Supplementary Figures S2 and S3) and day 8 (Figure 6). Mean peak areas of all metabolites and results of main effects are provided in Supplementary Table S5. Following are the significant differences at day 8 per linear mixed model analysis. Of the 32 ceramides/sphingomyelins detected, one was higher (Cer(d40:1)) and five were lower (SM(d32:1), SM(d32:2), SM(d36:1), SM(d36:2), SM(d38:2)). Twenty-three phosphatidylcholines (PC) detected, of these four were higher (PC(16:0_18:2), PC(16:1_18:2), PC(18:1_18:2), PC(20:0_18:2)) and three were lower (PC(18:0_22:5), PC(p-38:4)/PC(o-38:5), PC42:10). Interestingly, PCs that were statistically higher with the Intralipid infusion had less than four unsaturated bonds, whereas all PCs that lower had more than four unsaturated bonds. Of the 19 phosphatidylinositols (PI) detected, 4 were higher (PI34:2, PI36:3, PI36:4, PI38:5) and 3 were lower (PI30:0, PI40:4, PI40:6). Thirty-four phosphatidylethanolamines (PE) detected, of these four were higher (PE36:3, PE(16:0_18:2), PE(16:1_22:5)) and one was lower (PE38:5). Of the 21 phosphatidylserines (PS) detected, three were higher (PS34:2, PS34:3, PS36:3), and four were lower (PS32:1, PS34:1, PS38:4, PS38:5). Seventeen unbound fatty acids were detected, of these eight were higher (FA16:0, FA18:0, FA18:2, FA18:3, FA20:0, FA20:3, FA22:0, FA24:0). Of the 11 lysophosphatidylcholines (LPC) detected, 9 were higher (LPC16:0, LPC17:0, LPC18:0, LPC18:1, LPC18:2, LPC20:0, LPC20:1, LPC20:4, LPC24:0) and one was lower (LPC14:0). Of the three lysophosphatidylinositols (LPI) detected, two were higher (LPI18:0, LPI20:4). Three lysophosphatidylethanolamines (LPE) were detected, of these two were higher (LPE16:0, LPE18:0).

### Circulating fetal hormones

Hormones circulating in fetal plasma are shown in Figure 3 and Supplementary Table S1, and statistical analysis is provided in Tables 1 and Supplementary S2. Insulin levels were similar between treatment groups, and within the control (but not Intralipid) group increased between days 0 and 8 (*P*=0.018). Circulating IGF-1 decreased within the Intralipid group between days 0 and 8 (*P*<0.001). Independent of treatment, IGF-2 levels decreased slightly between days 0 and 8 (*P*=0.003). Although there was a significant interaction between treatment and day for plasma norepinephrine (*P*=0.037), no differences were significant by multiple comparisons.

### Fetal liver function

**Figure 7:**
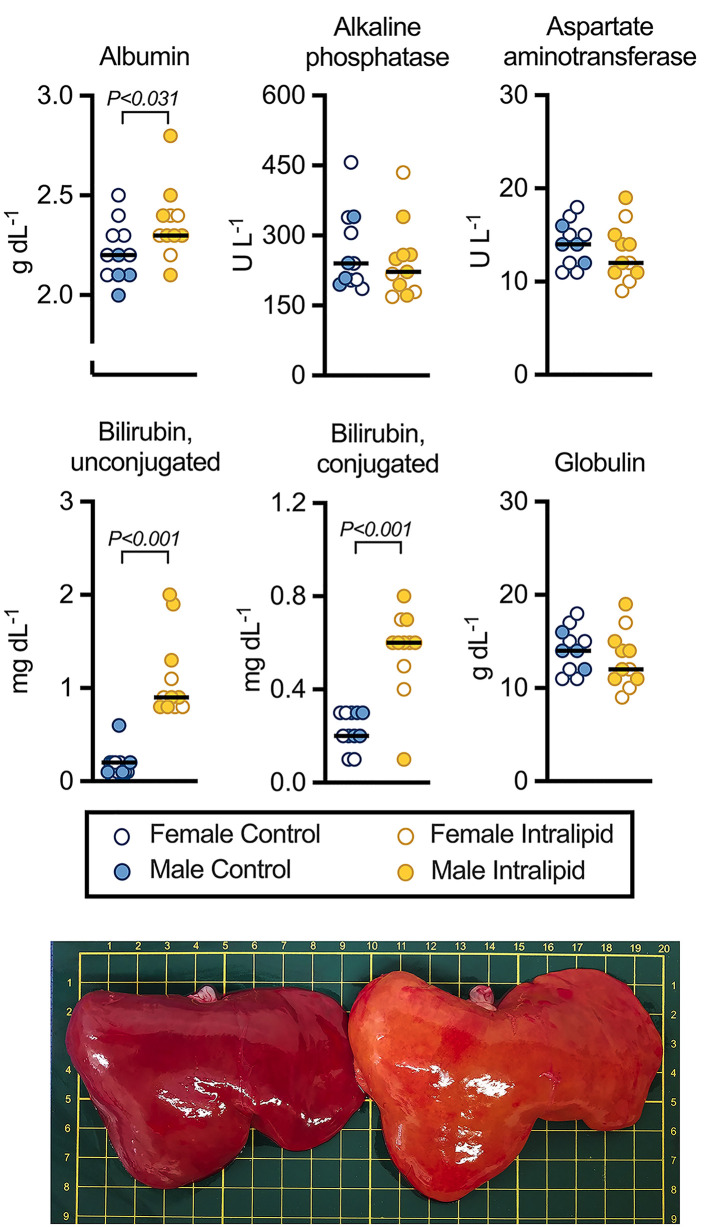
Fetal liver function panel results following Intralipid infusion Top: A panel of substances indicative of liver health were measured in fetal plasma on the terminal day. Liver damage may be indicated by elevated bilirubin, alkaline phosphatase, and aspartate aminotransferase, and by reduced albumin and globulin. Number for control females = 7, males = 4; Intralipid females = 4, males = 7. Individual data points shown with bar representing mean (sexes combined). Two-way ANOVA (between factors by treatment and sex). For complete statistical analysis, see Table 2. Bottom: Representative livers from a control (left) and an Intralipid-infused (right) fetus. Each square is 1 cm.

Results from a panel of assays indicative of liver health at the end of the infusion period are shown in Figure 7 and Table 2 and Supplementary S4. Plasma albumin levels were higher in fetuses that received intravenous Intralipid (*P*=0.031). Levels of unconjugated bilirubin were almost 6-fold higher in Intralipid-infused fetuses (*P*<0.001). Levels of conjugated bilirubin, which has been processed by the liver, were 2.5-fold higher than in controls (*P*<0.001). Alkaline phosphatase, aspartate aminotransferase, and globulin levels were not shown to be affected by fetal Intralipid infusion.

### Oil Red O staining

**Figure 8:**
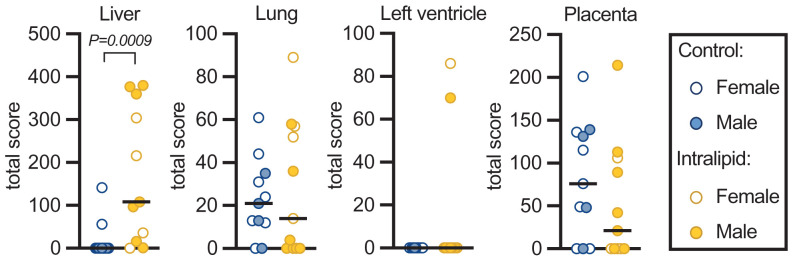
Oil Red O staining in fetal tissues Accumulated neutral lipids and lipid droplets within parenchymal cells were stained with Oil Red O in fetal liver, lung, heart, and placental tissues. Raw and median values are shown. Number for control females = 7, males = 4; Intralipid females = 4, males = 7. Treatment effect was visually assessed by sex prior to analysis by Mann–Whitney test.

Neutral lipid and lipid droplet content were assessed by Oil Red O staining. Staining in parenchymal cells of fetal liver was elevated 9-fold by Intralipid administration (*P*=0.0009; Figure 8). Lipid staining in fetal lung, heart and placenta were unaffected by treatment.

## Discussion

In the present study, we found that preterm fetal sheep (85–90% gestation) display tolerance to high plasma lipid levels occurring due to PN administration of Intralipid. Infusion had no effect on hemodynamic pressures or most arterial blood chemistry parameters, although it did depress circulating IGF-1 levels. There were considerable changes in the lipid constituents of RBCs, which suggests that lipid-dependent cellular functions throughout the body may be altered. Plasma protein and albumin levels were slightly elevated, suggesting intact liver function. Bilirubin levels accumulated, potentially due to increased RBC turnover and/or both limitations on the rate of unconjugated bilirubin elimination by the placenta. The liver was the only organ with gross changes, becoming blanched, and containing much more neutral lipid content.

### Lipid intolerance

Preterm and small for gestational age infants have lower lipid clearance rates of intravenous lipids than do older children and adults, suggesting impaired triglyceride hydrolysis and compromised cellular uptake and/or utilization of free fatty acids as fuel [25,26]. The associated elevation in plasma triglyceride levels above 200 or 250 mg dL^−1^ is considered evidence of lipid intolerance [27,28]. Factors associated with increased risk for lipid intolerance include sepsis, birth weight <1000 g, lipid emulsion PN >2.6 g kg^−1^ d^−1^, and gestational age <28 weeks [1,27]. The only risk factor present in this study was the high lipid emulsion infusion rate of 3 g kg^−1^ d^−1^, as the approximate human-equivalent age of our experimental animals at baseline was 33 weeks (35 weeks at necropsy). Although plasma triglyceride levels increased some 25-fold with PN in this study, the levels reached are similar to those of premature infants of similar developmental age given continuous lipid infusion [1], and do not indicate lipid intolerance. This suggests that this developmental age, or fetal physiology itself, for instance relatively low arterial oxygen and glucose levels and the presence of a placenta, is unlikely to increase risk for lipid intolerance.

Fat accumulation in the lungs has been described in pathology reports following death of infants that received Intralipid. In these cases, capillary engorgement with lipid was visible, with concentration of linoleic acid (indicating lipid emulsion origin), and involvement of tissue-resident macrophages in infants in whom infusion had been stopped several days prior [4]. In contrast, Intralipid infusion did not cause lipid accumulation in the parenchymal cells of the fetal sheep lungs, heart or placenta. Differences between those infants (mean of 29 weeks of gestation) and the animals in the present study include the level of development, the rate of lipid infusion (all infants had brief periods of infusion above the recommended rate), dependence on air breathing versus the placenta, and the presence of other complications of premature birth in the infants. Lipid accumulation in the lung did not occur in the present study, at a developmental age when sheep are ending the saccular stage of lung development and beginning alveologenesis [29]. However, many preterm infant lungs are in the late canalicular or early saccular stages, and we do not know whether fetal sheep lungs at these earlier stages of development would have reacted similarly.

The fetal heart primarily uses glucose and lactate oxidation for energy, whereas after birth lipid oxidation is the primary source [30–32]. In the mature heart, despite the reliance on lipids for energy, excess lipids contribute to pathology [33–36]. Much less is known about the relationship between lipids and myocardial health in the immature heart. Even brief exposure to high lipid levels in infancy is associated with increased aortic root diameter, aortic stiffness, and decreased peak systolic circumferential strain in later life [5,6]. Although there was normal Oil Red O staining in the left ventricular myocardium of the sheep in the present study, further investigation is warranted to determine the mechanisms contributing to cardiovascular pathology in individuals that received lipid emulsion PN.

In contrast with the lungs and heart, fetal liver lipids by Oil Red O increased >9-fold, with visible effects on organ coloration. These data indicate the relative sensitivity of the fetal liver to high lipid exposure, and a potential risk for PNALD. Associated with development of PNALD is cholestasis and failure to thrive [8]. Plasma protein (and albumin) levels increased in fetal sheep given Intralipid, which indicate an adaptive response by the liver. While there was an increase in both conjugated (direct) and unconjugated (indirect) bilirubin, the fold change was much greater for the unconjugated form, which has not yet been processed by the liver. This is expected, given the unique processing of bilirubin in the fetus compared to the neonate or adult, reflecting appropriately low fetal liver uridine-diphosphoglucuronic glucuronosyltransferase activity [37,38]. Bilirubin binds easily to albumin [39], and after birth it is processed in the liver to be excreted into bile. In contrast, bilirubin in the fetus is passed through the placenta into the maternal circulation. Consequently, elevated unconjugated bilirubin in the Intralipid-infused fetuses may represent impaired placental transfer or may reflect increased fetal RBC turnover. Although the fetal liver was clearly affected by lipid emulsion PN, there does not appear to be the onset of PNALD.

### Changed lipid concentrations in fetal RBCs

RBC phospholipids have been described as a stable measure of tissue fatty acid composition in parenterally fed preterm infants [40]; therefore, we measured fetal RBC lipids to understand lipid disposition in Intralipid-treated fetal sheep. The flexibility of the RBC membrane in folding and traversing capillaries is determined by the ratio of cholesterol to other lipids, particularly poly unsaturated fatty acids [41]. RBCs are a cell type that is relatively homogeneous as a population and do not require complex procedures to gain purity. For this reason, they are a minimally invasive surrogate marker for demonstrating that lipid infusion had cellular effects and can provide particular insight when compared with other lipid therapy modalities [11]. Changes in RBC lipids reflects changes in the constituents in lipid membranes of other cells [42–45]. The changes described above for cellular functions dependent on lipid composition will affect the function of other organs. Cell-specific differences we were unable to measure in the present study will occur as well, for instance, membrane lipid composition of cardiomyocytes affects ion channel function [46].

Unsurprisingly, Intralipid components derived from soy oil and egg phospholipids are increased in infused fetuses, notably linoleic acid which comprises 44–62% of Intralipid [47,48]. Treatment increased unbound fatty acid levels broadly, especially the saturated fatty acids despite Intralipid being comprised primarily of unsaturated fatty acids [48]. In particular, palmitic acid (16:0), the first fatty acid produced during fatty acid synthesis and a major component of mammalian cells and Intralipid (comprising 7–14%), and stearic acid (18:0), another very common fatty acid in the body and in Intralipid (comprising 1.4–5.5%), were increased [48]. Interestingly, oleic acid comprises 19–30% of Intralipid but was not increased in Intralipid-treated versus control fetal RBCs. These changes indicate that lipids taken up by fetal tissues (RBCs) not only reflect the composition of Intralipid but also indicate modifications to infused lipids in that they are more saturated and that oleic acid is not increased. Notably, neonatal RBCs are stiffer and already containly slightly more saturated fatty acids compared with adult RBCs, and these changes are likely to increase rigidity further [49,50].

PC is a major constituent of lipid bilayers and is readily available in both soy and egg yolk; Intralipid comprises 1.2% egg phospholipids [51]. PS is also a component of cell membranes and plays a role in cell cycle and apoptosis signaling. PI is a minor constituent of lipid bilayers. Decreased phospholipid fatty acid tail lengths tend to reduce the freezing point of lipid bilayers, making them more fluid [52]. Among these two fatty acid-bound phospholipids, species increased by Intralipid tended to have fewer total acyl carbons, and species decreased by Intralipid tended to have more total acyl carbons.

PE is a non-bilayer forming phospholipid which is, nevertheless, the second most abundant glycophospholipid in the cell [53]. PE is the substrate for synthesis of PC, thus the increased concentration of PE with fewer than 40 total acyl carbons following Intralipid treatment potentially explains the rise in PCs with a similar length of total acyl carbons. Other notable roles for PE include assisting in membrane fusion at the cleavage furrow during cytokinesis, facilitating oxidative phosphorylation through organization of complex IV, mitochondrial biogenesis, and autophagy [53].

Lysophospholipids are bioactive signaling lipids derived from membrane phospholipids and sphingolipids by phospholipases, and many were increased in fetal RBCs following Intralipid treatment. LPI is a second messenger which modulates calcium-dependent cell processes in the pancreas, smooth muscle, heart and other tissues [54]. LPE is a minor constituent of cell membranes with signaling properties that may suppress lipolysis and fatty acid biosynthesis [55]. LPC is a minor constituent of cell membranes, sometimes serves as a signal to stimulate phagocytosis (as of apoptotic cells) and is generally short-lived due to metabolism by lysophospholipase and LPC-acyltransferase. As 9/10 LPC species were increased by Intralipid treatment, apoptotic signaling in treated fetuses may be increased.

Ceramides are the component lipid of sphingomyelins, and sphingomyelins are a major constituent of the cell membrane lipid bilayer. Many ceramides/sphingomyelins decreased in response to Intralipid treatment of fetal sheep. As sphingomyelins are critical components of lipid rafts, which are important for endocytosis, exocytosis and cell signaling, decreased concentrations may impair these cell functions [52].

### Dose of Intralipid

Clinical guidelines written for human administration were used to guide dosage of this study, but species-specific differences which may influence how Intralipid is processed and consequently the effects of a particular dose. However, supporting use of a similar per kilogram dose in sheep is the similarity between the nutrition recommended for premature infants and nutrient transfer rates across the placenta in the fetal sheep [56–58]. It is stated that lipids should provide 30–50% of nonprotein energy, which in premature infants helps to optimize protein accretion while limiting the potential toxicity of excessive lipids [56]. In sheep fetuses at ∼131 dGA (∼3 kg), umbilical glucose uptake is 99 μmol min^−1^ (∼33 μmol min^−1^ kg^−1^), and umbilical lactate uptake is 49 μmol min^−1^ (16 μmol min^−1^ kg^−1^) [57]. The relationship between glucose and lactate uptake by the fetus is linear over a range of fetal weights spanning 2.25–5 kg [58]. As carbohydrates yield approximately 17 kJ g^−1^, glucose is 180 g mol^−1^, and lactate is 90 g mol^−1^, fetal sheep receive approximately 6.1 kJ kg^−1^ h^−1^ from glucose and 1.5 kJ kg^−1^ h^−1^ from lactate, for a total of 7.6 kJ kg^−1^ h^−1^. The recommendation of a maximum of 30–50% of energy from intravenous fatty acid emulsion would yield 37 kJ g^−1^ of fat (1.5–2.5 g kg^−1^ h^−1^) in this context. Our maximal rate of infusion, 2.8 ± 0.5 g kg^−1^ d^−1^, therefore theoretically exceeds the recommended maximum for lipid-derived energy for preterm infants by 12–87%.

### Intralipid versus other forms of lipid emulsion parenteral nutrition

A consideration in lipid emulsion PN is necessity of supplying sufficient essential fatty acids: ω-6 polyunsaturated fatty acid (PUFA) linoleic acid, and ω-3 PUFA α-linolenic acid [59]. Linoleic acid comprises approximately 50% of soybean oil and α-linolenic acid approximately 4–11% [48,59]. However, soybean oil does not contain either of the very long chain ω-3 PUFAs, eicosapentaenoic acid (EPA), and docosahexaenoic acid (DHA), which have critical roles in early retinal and brain development. There are further concerns that the high amounts of linoleic acid in soybean oil also impair the synthesis of these very long chain ω-3 from the small amount of available α-linolenic acid. Unsurprisingly, RBCs of fetuses that received Intralipid in this study had elevated levels of unbound fatty acids, FA18:2 and FA18:3, which likely indicate an enrichment of linoleic and α-linolenic acid, respectively (the assay does not distinguish between ω-3 and ω-6). Notably, however, there were no decreases in fetal RBC levels of EPA (20:5) or DHA (22:6), nor increases in arachidonic acid (AA; 20:4), into which linoleic acid is converted. Maintenance of DHA and AA may be due to concentration by the placenta from maternal blood [60].

Soybean based-lipid emulsion PNs are regarded as having a potential inflammatory-stimulating role, perhaps due to their imbalanced levels ω-6 and ω-3 PUFAs, and for contributing to hepatic steatosis [59]. Fish-oil based lipids have advantages with regards to liver pathology [61]. Our findings of substantially increased lipid staining of the liver (Figure 8) contribute to our concern about use of Intralipid in preterm infants.

Although soybean-based lipid emulsions such as Intralipid remain the most commonly used [61] lipid emulsion PE, newer formulas containing mixtures of fish and olive oil lipids are gaining ground due to their many advantages. It is not known how newer generation lipid emulsions would affect essential fatty acid levels or liver lipids in our model.

### Nutrient and hormonal milieu

IGF-1 is a critical signal of fetal nutritional status and regulator of fetal growth [62], and in the present study, Intralipid decreased IGF-1 levels by 30%. In fetal sheep given exogenous IGF-1 for a week, insulin and glucose levels are diminished [63]. Despite the decreased IGF-1 in fetuses receiving Intralipid, glucose and insulin levels did not change. Our study was not designed to measure slowed growth, which requires extended experimental timelines or large study groups [24,64,65]. Consequently, we cannot tell if the reduced IGF-1 levels would eventually impact somatic growth in fetuses receiving PN Intralipid. Notably, it has recently been reported that newborns affected by fetal growth restriction had elevated cord blood triglyceride levels [66]. The authors posited that impaired placental lipid trafficking occurred as a consequence of the etiology causing fetal growth restriction but speculate that this may impact fetal growth. These findings may also raise further concern for preterm neonates given PN lipid, as IGF-1 levels fall and are low following preterm birth [67].

### Sex differences

Although differences between male and female fetal lipid transporters and blood levels have been described in the context of maternal obesity [65], Intralipid infusion did not appear to affect fetal hemodynamics, blood chemistry or organ growth differently on the basis of sex (with the possible exception of glucose). There were some differences between females and males irrespective of treatment. In male fetuses, but not females, there was a 1.5 mmHg increase in arterial pressure between day 125 ± 1 and 133 ± 1 dGA. We did not find that males had higher arterial pressure than females, and such a difference is not reported in other experimental studies [68–70]. Male fetuses weighed 600 g more than female fetuses at the end of the study period, irrespective of treatment, a sex difference that is not typically observed in small experimental studies [14,71–73]. Male livers were 29 g (4 g kg^−1^) heavier than female livers.

### Limitations of the study

While we chose to study fetuses rather than preterm lambs in order to experimentally isolate the effects of PN Intralipid 20® from the myriad of physiological changes associated with preterm birth, the present study is limited in that it does not include those impacts and their potential influence on response to lipid infusion, and that arterial PO_2_ is much lower in fetuses than neonates. The fetuses included in the present study were equivalent to moderately preterm infants, and so we expect them to have more mature capacity for lipid uptake, storage, conversion and metabolism than very preterm (<32 weeks) infants. It would be valuable to understand the response to Intralipid in this model at an extremely preterm equivalent age, which would be less than approximately 105 dGA in sheep. Another limitation is that we only studied the effects of Intralipid 20®. While still the most commonly used lipid emulsion PN, newer generation lipid emulsions are considered by many as superior. How these emulsions based on olive and fish oils would differently affect the fetus is unclear and would be intriguing to study. We also used an untargeted lipidomics assay which does not provide complete lipid resolution. Although this assay can distinguish between lipid classes and provide some structural resolution (e.g., total acyl carbons, number of double bonds in fatty acids, etc.), it generally lacks positional and orientation information of double bonds, fatty acid constituents, stereochemistry, and fatty acid position on glycerol backbone. This greater level of detail would improve our ability to interpret activity in lipid synthetic and metabolic pathways.

## Clinical perspectives

- Parenteral Intralipid nutrition may contribute to pathology observed in some preterm neonates, yet understanding the contribution of Intralipid versus the other physiological impacts of preterm birth is challenging; therefore, we studied Intravenous treatment of the near-term fetal lamb.
- Intravenous lipid infusion altered the metabolic milieu of the fetus, changing relative concentrations of lipids important for cell signaling and function within the plasma and RBCs, causing lipid accumulation in the liver (but not heart, lung or placenta), and decreasing the circulating levels of growth hormone IGF-1.
- At 85–90% gestation, the fetus is relatively tolerant of intravenous lipid emulsion, although accumulation of lipid in the liver may indicate it is at particular risk at this developmental stage, and reduced IGF-1 levels may complicate the challenges in achieving ideal growth faced by preterm neonates.

## Supplementary Material

Supplementary Figures S1-S3 and Tables S1-S5

## Data Availability

Data are available upon reasonable request of the corresponding author.

## Abbreviations

dGA: days of gestational age
IGF-1: insulin-like growth factor 1
IGF-2: insulin-like growth factor 2
IGFBP1: insulin-like growth factor binding protein 1
IGFBP2: insulin-like growth factor binding protein 2
LPC: lysophosphatidylcholine
LPE: lysophosphatidylethanolamine
LPI: lysophosphatidylinositol
PC: phosphatidylcholine
PCO_2_: arterial partial pressure of carbon dioxide
PE: phosphatidylethanolamine
PI: phosphatidylinositol
PN: parenteral nutrition
PNALD: PN-associated liver disease
PO_2_: arterial partial pressure of oxygen
PS: phosphatidylserine
RBC: red blood cell
