# Correction: Physiological response to fetal intravenous lipid emulsion

**DOI:** 10.1042/CS20231419_COR

**Published:** 2025-05-20

The authors of the research article “Physiological response to fetal intravenous lipid emulsion” (10.1042/CS20231419) would like to correct their paper.

The Oil Red O staining reported within this paper was performed by a different laboratory (as noted in the acknowledgements). On using a different laboratory to the original one for Oil Red O staining in subsequent studies, a different staining pattern to that published in the *Clinical Science* paper has been discovered. Following further investigation, the authors would like to correct the sections noted below in the published article. The Editor-in-Chief and Editorial Board approve the correction. The authors apologise for any inconvenience caused.

## Abstract

**Original text:** Compared with control Oil Red O liver stains (median score 0), Intralipid-infused fetuses scored 108 (P=0.0009).

**Corrected text:** Compared with control Oil Red O, liver, lung, and cardiac tissues contained significantly more neutral lipid content (2-, 4-, and 39-fold respectively).

## Methods

### Oil red O staining

Tissues were frozen in OCT (Optimal Cutting Temperature Media, Fisher Sci, cat: 23–730-571) and sectioned at 10 μm on a Leica CM3050S cryostat. Tissue sections from experimental animals and positive controls were mounted onto Leica Apex clipped corner microscope slides and kept at -80°C until staining. To stain for Oil Red O, slides were brought to room temperature for 15 minutes and fixed in 10% Neutral Buffered Formalin for 1 minute. Slides were incubated in double-filtered Oil Red O solution (0.5% w/v Oil Red O in isopropanol and diluted 3:2 in distilled water) for 10 minutes and rinsed in tap water. They were then counterstained with hematoxylin (Epic Scientific, cat: HEM-C with 4.17% v/v glacial acetic acid) for 1 minute, incubated in bluing (Epic Scientific, BLU-C) for 1 minute, and mounted with 1:1 diluted Clear-Mount (Electron Microscopy Sciences cat: 1798515) until dry. After dipping in xylenes, slides were coverslipped on a Leica CV5030 automatic coverslipper. Images were captured using a Leica AT2 slide scanner (Leica Biosystems Imaging, Inc.) controlled by Leica ScanScope Scanner Console (Version 102.0.7.5), using an Olympus Plan Apo 20 x / 0.75 NA air objective with a 503.1 nm/pixel XY resolution. Images were saved in Tiled TIFF format (SVS) for image analysis. Images were coded to obscure treatment groups. Low power scan was used to confirm relative uniformity of staining. Images were scored by a pathologist (AC) according to criteria developed and validated to diagnose milk aspiration in pulmonary macrophages from pediatric bronchoalveolar lavage samples [20]. Scoring was performed using Leica ImageScope (Version 12.4.6.5003) in 100 parenchymal cells at the “20 x” setting in a random but representative field, then in another 100 cells in 1–3 more non-contiguous random but representative fields (avoiding tissue edges and folded tissues). Scores were summed within each field and averaged between fields. Number of scored tissues is reduced by tissues lost after initial analysis.

## Results

### Oil red O staining

Neutral lipid and lipid droplet content were assessed by Oil Red O staining. Staining in parenchymal cells of fetal liver was elevated 2-fold by Intralipid administration (*P*=0.0125), staining in the lung was elevated 4-fold (*P*=0.0318), and in the left ventricle it was elevated 39-fold (*P*=0.0002; Revised Figure 8). Placental staining was not increased (*P*=0.1205). Periodic Acid-Schiff staining was used to distinguish maternal from fetal cells in the placenta; placental staining was predominantly in maternal cells.

**Figure 8:**
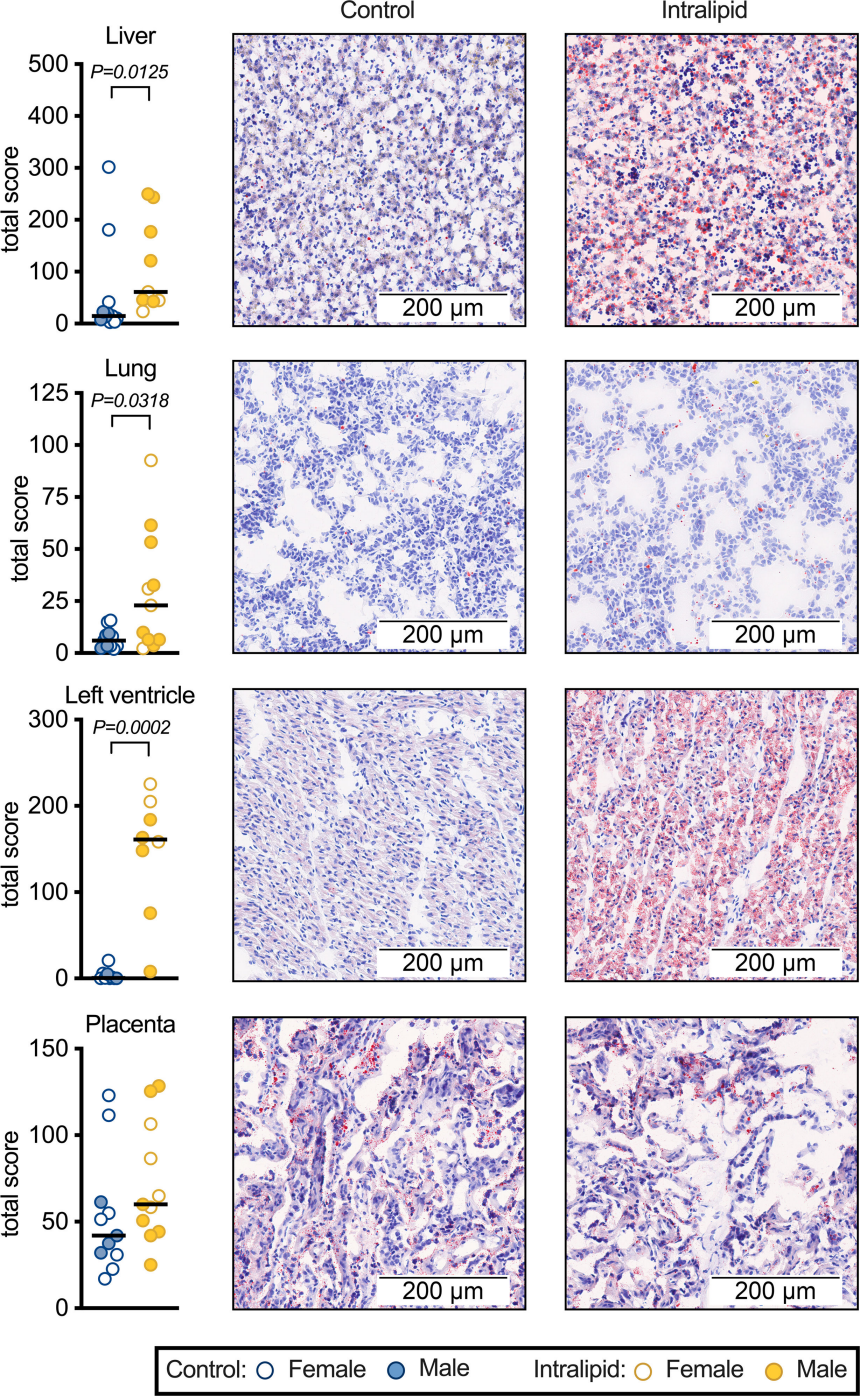
Oil Red O staining in fetal tissues Accumulated neutral lipids and lipid droplets were stained with Oil Red O in fetal liver, lung, heart, and placental tissues. Raw and median values are shown. Number for control females = 7 (except LV = 6), males = 4 (except LV = 3); Intralipid females = 4 (except liver and LV = 3), males =7 (except liver = 6, LV = 5). Treatment effect was visually assessed by sex prior to analysis by Mann–Whitney test.

## Discussion: first paragraph

**Original text:** The liver was the only organ with gross changes, becoming blanched, and containing much more neutral lipid content.

**Corrected text:** The liver becoming blanched, and all organs assessed, except placenta, were found to contain much more neutral lipid content.”

## Discussion: Lipid intolerance section

Preterm and small for gestational age infants have lower lipid clearance rates of intravenous lipids than do older children and adults, suggesting impaired triglyceride hydrolysis and compromised cellular uptake and/or utilization of free fatty acids as fuel [25,26]. The associated elevation in plasma triglyceride levels above 200 or 250 mg dL^-1^ is considered evidence of lipid intolerance [27,28]. Factors associated with increased risk for lipid intolerance include sepsis, birth weight < 1000 g, lipid emulsion PN>2.6 g kg^-1^ d^-1^, and gestational age<28 weeks [1,27]. The only risk factor present in this study was the high lipid emulsion infusion rate of 3 g kg^-1^ d^-1^, as the approximate human-equivalent age of our experimental animals at baseline was 33 weeks (35 weeks at necroscopy). Although plasma triglyceride levels increased some 25-fold with PN in this study, the levels reached are similar to those of premature infants of similar developmental age given continuous lipid infusion [1], and do not indicate lipid intolerance. This suggests that this developmental age, or fetal physiology itself, for instance relatively low arterial oxygen and glucose levels and the presence of a placenta, is unlikely to increase risk for lipid intolerance.

Fat accumulation in the lungs has been described in pathology reports following death of infants that received Intralipid. In those cases, capillary engorgement with lipid was visible, with concentration of linoleic acid (indicating lipid emulsion origin), and involvement of tissue-resident macrophages in infants in whom infusion had been stopped several days prior [4]. In contrast, Intralipid infusion caused lipid accumulation primarily in the parenchymal cells of the fetal sheep lungs (accumulation in histiocytes was not excluded). Differences between those infants (mean of 29 weeks of gestation) and the animals in the present study include the level of development, the rate of lipid infusion (all infants had brief periods of infusion above the recommended rate), dependence on air breathing versus the placenta, and the presence of other complications of premature birth in the infants. Further, the fetal sheep in this study were ending the saccular stage of lung development and beginning alveologenesis [29], while many preterm infant lungs are in the late canalicular or early saccular stages. We do not know whether fetal sheep lungs at these earlier stages of development would have reacted similarly.

The fetal heart primarily uses glucose and lactate oxidation for energy, whereas after birth lipid oxidation is the primary source [30-32]. In the mature heart, despite the reliance on lipids for energy, excess lipids contribute to pathology [33-36]. Much less is known about the relationship between lipids and myocardial health in the immature heart. Even brief exposure to high lipid levels in infancy is associated with increased aortic root diameter, aortic stiffness, and decreased peak systolic circumferential strain in later life [5,6]. Although Oil Red O staining was augmented in the left ventricular myocardium of the Intralipid-infused sheep in the present study, further investigation is warranted to determine the mechanisms contributing to cardiovascular pathology in individuals that received lipid emulsion PN.

Fetal liver lipids by Oil Red O increased 2-fold, with visible effects on organ coloration. These data indicate the relative sensitivity of the fetal liver to high lipid exposure, and a potential risk for PNALD. Associated with development of PNALD is cholestasis and failure to thrive [8]. Plasma protein (and albumin) levels increased in fetal sheep given Intralipid, which indicate an adaptive response by the liver. While there was an increase in both conjugated (direct) and unconjugated (indirect) bilirubin, the fold change was much greater for the unconjugated form, which has not yet been processed by the liver. This is expected, given the unique processing of bilirubin in the fetus compared to the neonate or adult, reflecting appropriately low fetal liver uridine-diphosphoglucuronic glucuronosyltransferase activity [37,38]. Bilirubin binds easily to albumin [39], and after birth is processed in the liver to be excreted into bile. In contrast, bilirubin in the fetus is passed through the placenta into the maternal circulation. Consequently, elevated unconjugated bilirubin in the Intralipid-infused fetuses may represent impaired placental transfer or may reflect increased fetal RBC turnover. Although the fetal liver was clearly affected by lipid emulsion PN, there does not appear to be the onset of PNALD.

## Addition to Acknowledgements

The authors thank Ms Rachel Dannay and Dr Sathya Srinivasan of the Integrated Pathology Core and advanced optical microscopy facility at Oregon National Primate Research Center, which is supported by NIH grant P51OD011092. The Leica AT2 scanner was supported by NIH grant S10OD025002.

