# Supplementary material for: Physiological response to fetal intravenous lipid emulsion
Source: Clin Sci (Lond). 2024 Feb 2;138(3):117–34. doi: 10.1042/CS20231419 (PMC10876438; doi:10.1042/CS20231419)
Supplement: Supplementary Figures S1-S3 and Tables S1-S5 [file CS-2023-1419_supp.pdf]

Physiological response to fetal intravenous lipid emulsion

Supplementary Material

Brian D. Piccolo[1,2], Athena Chen[3, 4], Samantha Louey[4], Kent L.R. Thornburg[4], Sonnet S. Jonker[4]

[1]USDA/ARS-Arkansas Children's Nutrition Center, Little Rock, AR, USA.
[2]Department of Pediatrics, University of Arkansas for Medical Sciences, Little Rock, AR, USA.
[3]Department of Pathology, Oregon Health & Science University, Portland, OR, USA.
[4]Center for Developmental Health, Knight Cardiovascular Institute, Oregon Health & Science University, Portland, OR, USA.

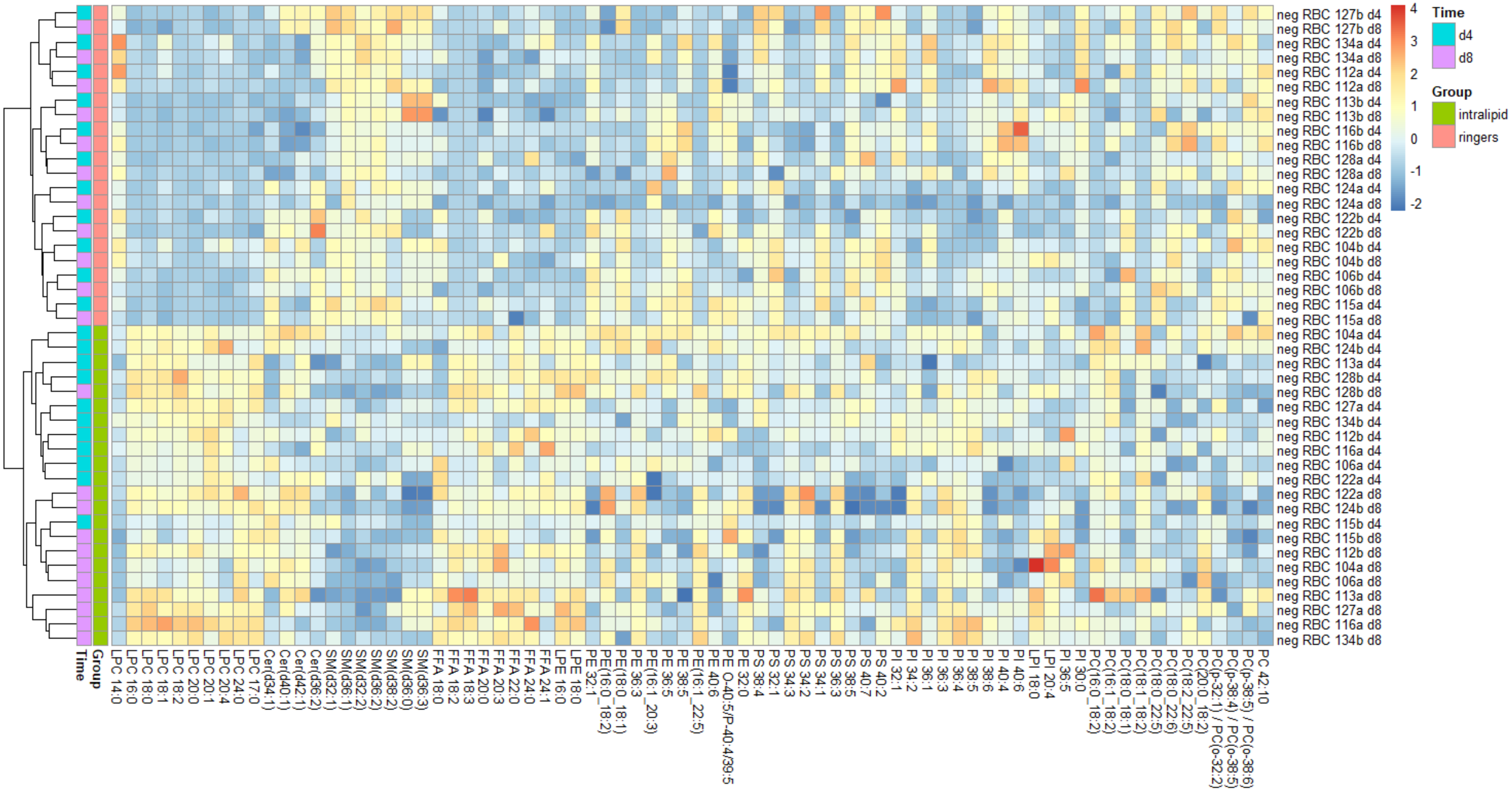

Figure S1. Heat map of lipid profiles of fetal red blood cells from experimental days 4 and 8

The order of metabolite columns is maintained within their respective classes, while the order of the rows is based on the clustering algorithm which orders the samples based on the similarities. This display of the RBC lipid data show that the Intralipid-treated fetuses cluster from the Lactated Ringer's Solution-treated Controls, that the Intralipid-treated group is mainly separated by time, and that within the Control group the individual animals are similar between days.

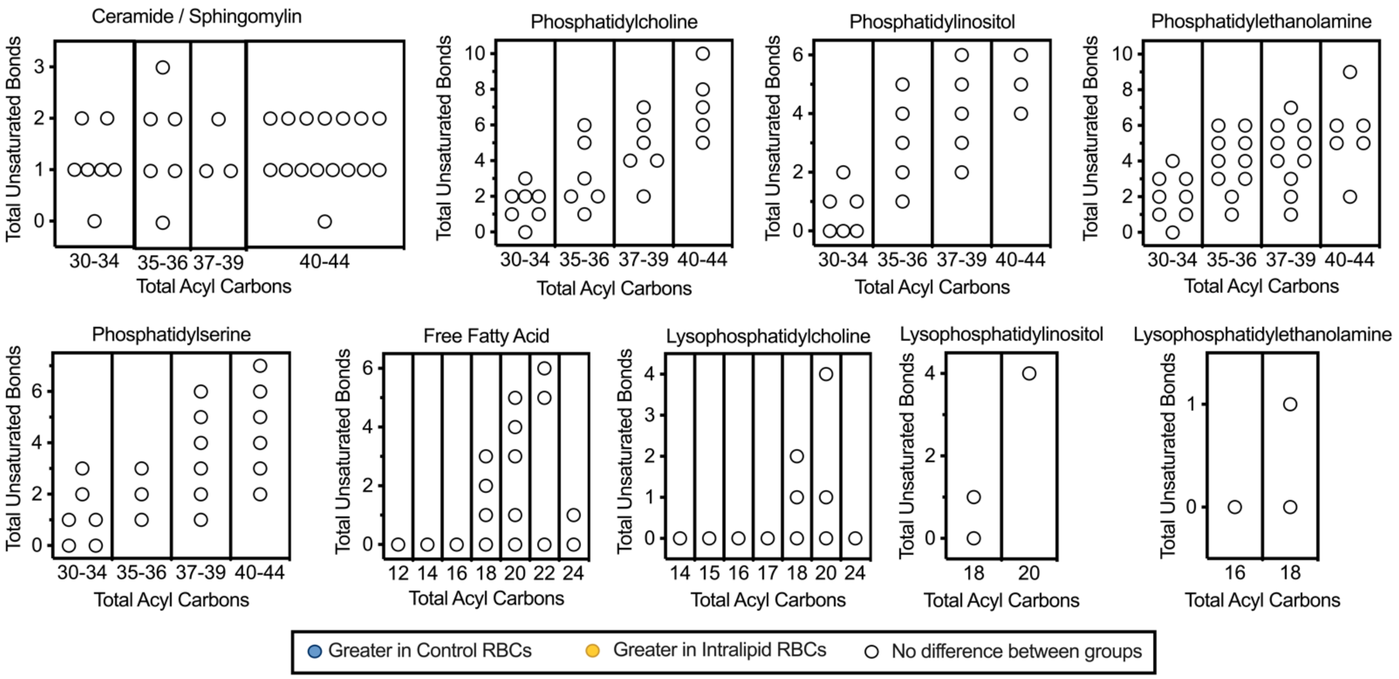

Figure S2. Lipid profiles of fetal red blood cells before treatment (day 0)

Relative concentrations of lipids within fetal RBCs were determined by LC-MS. There were no differences at day 0 between the Control and Intralipid 20-treated groups. Univariate analysis was performed in a linear mixed model 2x3 approach (experimental groups by time points) with individual fetus as a random effect (data from days 0 and 4 are in the Supplement). All main effects and interaction terms are adjusted for multiple comparisons using the Benjamini and Hochberg false discovery rate correction.

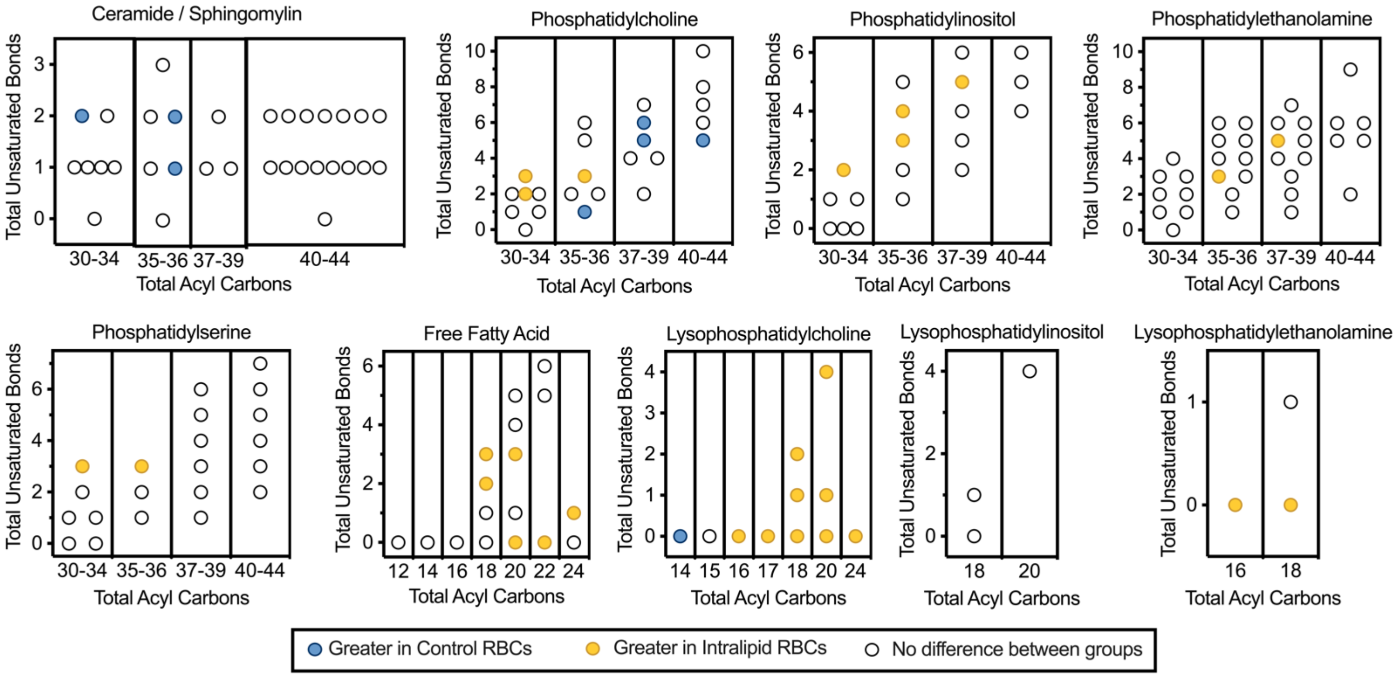

Figure S3. Lipid profiles of fetal red blood cells after 4 days of treatment

Relative concentrations of lipids within fetal RBCs were determined by LC-MS. By the fourth day of intravenous Intralipid 20, 28 of the 163 detected lipid species were elevated, and 8 were depressed compared to Control fetuses. Univariate analysis was performed in a linear mixed model 2x3 approach (experimental groups by time points) with individual fetus as a random effect (data from days 0 and 4 are in the Supplement). All main effects and interaction terms are adjusted for multiple comparisons using the Benjamini and Hochberg false discovery rate correction.

Table S1. Mean and standard deviations from analysis of arterial lipids by sex

		Day 0		Day 4		Day 8	
		Control	Intralipid	Control	Intralipid	Control	Intralipid
**Log Cholesterol, plasma**	**Female**	1.21±0.09	1.19±0.08	1.28±0.07	1.68±0.20	1.24±0.09	1.57±0.18
	**Male**	1.18±0.11	1.14±0.11	1.23±0.12	1.56±0.13	1.20±0.04	1.46±0.15
**Log Phospholipids, plasma**	**Female**	1.34±0.14	1.32±0.07	1.35±0.05	1.74±0.08	1.39±0.05	1.84±0.11
	**Male**	1.31±0.15	1.30±0.08	1.38±0.06	1.65±0.18	1.36±0.06	1.75±0.11
**Log Triglycerides, plasma**	**Female**	0.28±0.13	0.40±0.07	0.31±0.12	1.88±0.24	0.28±0.14	1.77±0.21
	**Male**	0.29±0.12	0.41±0.2	0.24±0.09	1.62±0.36	0.28±0.12	1.55±0.45

Table S2. F statistics from analysis of hemodynamic and arterial blood chemistry parameters

	3-Way Interaction	2-Way Interactions			Main Effects		
		Test only if 3-way interaction is NS.			*Test only if 2- and 3-way interactions are NS.*		
	**Treatment ×Day×Sex**	**Treatment ×Day**	**Day ×Sex**	**Treatment ×Sex**	**Day**	**Treatment**	**Sex**
**Arterial pressure** (mmHg)	0.358	0.068	4.755	1.189	-	-	-
**Venous pressure** (mmHg)	0.000	0.045	1.051	0.240	2.301	0.052	1.172
**Heart rate** (bpm)	4.093	1.010	0.831	0.001	37.489	0.082	4.183
**pH**	0.147	0.105	0.005	1.427	0.093	0.954	0.102
**Hematocrit** (%)	0.001	2.753	1.086	0.800	0.743	0.028	0.014
**Total hemoglobin** (g dL^{-1})	0.019	1.177	0.064	1.329	0.938	0.768	0.187
**PCO_2** (mmHg)	0.624	0.130	0.611	2.225	1.745	0.012	0.614
**PO_2** (mmHg)	0.260	0.694	0.420	1.146	12.481	1.538	0.002
**O_2-Hb saturation** (%)	1.125	1.777	0.641	0.816	0.267	2.536	0.697
**O_2 content** (mL dL^{-1})	0.312	2.070	0.446	2.149	0.434	2.179	0.385
**Plasma protein** (g dL^{-1})	1.452	11.868	0.127	0.064	-	-	-
**Glucose** (mmol L^{-1})	4.606 *a*	-	-	-	-	-	-
**Lactate** (mmol L^{-1})	0.323	0.250	0.019	0.684	2.755	0.468	1.221
**Insulin** (ng mL^{-1})	0.265	5.729	0.745	0.010	-	-	-
**IGF-1** (ng mL^{-1})	3.433	10.861	1.042	0.145	-	-	-
**IGF-2** (ng mL^{-1})	0.034	1.462	0.014	0.580	5.344	0.065	0.628
**Norepinephrine** (pg mL^{-1})	0.344	5.100	0.019	0.020	-	-	-
**Log Cholesterol, plasma**	0.987	29.562	0.187	0.499	-	-	-
**Log Phospholipids, plasma**	2.266	15.965	0.026	0.492	-	-	-
**Log Triglycerides, plasma**	0.744	55.150	0.659	0.144	-	-	-

Number for Control female=7, male=4; Intralipid female=4, male=7; except number for pH Control female=3, male=3; Intralipid female=2, male=4. Mixed measures three-way ANOVA (3 levels of repeated measures for plasma lipids, 2 levels for all other variables) with the Greenhouse-Geisser correction for sphericity. Not significantly different (NS). Hemoglobin (Hb).

(a) F statistics for simple 2-way interaction following significant 3-way interaction using Bonferroni correction for multiple comparisons: Treatment×Day Female =1.714, Male =0.078.

Table S3. Mean and standard deviations from analysis of hemodynamic and arterial blood chemistry parameters by sex

	Day 0				Day 8			
	Control		Intralipid		Control		Intralipid	
	Female	Male	Female	Male	Female	Male	Female	Male
**Arterial pressure** (mmHg)	45.3±1.1	41.5±1.4	42.3±1.2	41.6±4.7	44.6±2.5	43.2±1.5	42.3±1.5	43.0±3.8
**Venous pressure** (mmHg)	3.1±1.5	2.0±0.3	2.9±0.5	2.2±0.7	3.3±1.9	3.1±2.5	3.3±0.6	3.5±1.2
**Heart rate** (bpm)	165±14	166±8	158±7	169±11	138±11	154±8	148±14	153±10
**pH**	7.4±0.02	7.383±0.022	7.374±0.022	7.385±0.023	7.385±0.019	7.385±0.014	7.376±0.006	7.38±0.021
**Hematocrit** (%)	35±8	33±3	35±5	36±5	35±7	34±1	32±7	35±5
**Total hemoglobin** (g dL^{-1})	11.0±1.5	10.5±0.5	10.3±1.9	11.3±1.6	11.0±1.8	10.6±0.4	9.8±2.6	10.9±1.7
**PCO_2** (mmHg)	51±3	52±4	54±4	50±2	51±4	52±1	52±3	50±3
**PO_2** (mmHg)	21±2	20±4	19±1	21±4	20±2	18±1	17±2	17±2
**O_2-Hb saturation** (%)	55±9	51±11	46±12	53±9	53±11	57±5	42±14	49±11
**O_2 content** (mL dL^{-1})	8.1±1.3	7.3±1.7	6.6±2.6	8±1.2	7.9±1.7	8.0±0.4	5.8±2.8	7.3±2
**Plasma protein** (g dL^{-1})	3.2±0.1	3.2±0.2	3.3±0.2	3.2±0.2	3.5±0.3	3.4±0.4	3.8±0.4	3.9±0.2
**Glucose** (mmol L^{-1})	1±0.2	1.1±0.2	1±0.3	1.1±0.1	0.9±0.2	1.2±0.0	1.2±0.3	1.2±0.2
**Lactate** (mmol L^{-1})	1.6±0.5	1.5±0.7	1.9±1	1.5±0.4	1.7±0.6	1.6±0.2	2.1±0.7	1.6±0.5
**Insulin** (ng mL^{-1})	0.4±0.15	0.48±0.27	0.42±0.17	0.52±0.30	0.49±0.20	0.69±0.41	0.36±0.09	0.49±0.16
**IGF-1** (ng mL^{-1})	60±23	77±35	55±17	73±26	000000	81±34	38±14	51±28
**IGF-2** (ng mL^{-1})	1.6±0.2	1.6±0.1	1.6±0.2	1.7±0.2	1.6±0.2	1.6±0.3	1.4±0.2	1.5±0.2
**Norepinephrine** (pg mL^{-1})	241±197	119±68	252±255	149±127	156±89	64±30	361±335	209±180

Table S4. F statistics, mean and standard deviation from analysis of fetal weights and liver function panel

	F statistic			Mean±SD			
				Control		Intralipid	
	Interaction	Treatment	Sex	Female	Male	Female	Male
**Body weight** (kg)	0.359	0.006	4.825	3.8±0.7	4.5±0.2	4.0±0.7	4.4±0.6
**Heart weight** (g)	0.126	1.100	1.126	26.7±5.5	28.1±3.7	28.1±2.1	30.9±4.8
**Liver weight** (g)	0.553	0.862	4.728	70.9±15.9	95.8±28.4	75.7±17.2	105.5±25.8
**Heart/body** (g kg^{-1})	0.584	1.679	1.270	7.0±0.7	6.2±0.7	7.2±1.3	7.1±1.1
**Liver/body** (g kg^{-1})	0.065	0.550	7.748	18.7±3.2	21.0±5.8	19.0±2.5	23.7±3.2
**Albumin** (g dL^{-1})	3.129	5.442	0.805	2.3±0.1	2.1±0.1	2.3±0.1	2.4±0.2
**Alkaline phosphatase** (U L^{-1})	0.086	0.161	0.255	277±98	246±65	250±125	242±54
**Aspartate aminotransferase** (U L^{-1})	0.555	0.950	0.398	14.1±2.9	14.0±1.6	12.0±3.6	13.7±2.8
**Bilirubin, unconjugated** (mg dL^{-1})	0.677	36.083	2.163	0.2±0.1	0.3±0.2	0.9±0.1	1.2±0.5
**Bilirubin, conjugated** (mg dL^{-1})	0.012	24.557	0.186	0.2±0.1	0.3±0.1	0.6±0.1	0.6±0.2
**Globulin** (g dL^{-1})	0.146	1.664	0.386	14.1±2.9	14.0±1.6	12.0±3.6	13.7±2.8

Table S5. Metabolite differences in fetal red blood cells

		Control			Intralipid			FDR corrected P-values		
Class	Metabolite	day 0	day 4	day 8	day 0	day 4	day 8	Interaction	Group	Time
Cer	Cer(d40:1)	179791 (9078)	191653 (9372)	186737 (11163)	191731 (6014)	215446 (10133)	244458 (8197)	6.08E-06	-	-
Cer	Cer(d34:1)	229428 (13174)	239070 (15959)	209629 (13122)	224295 (15921)	238566 (17076)	240984 (16463)	7.91E-03	-	-
Cer	Cer(d42:1)	233894 (11198)	252438 (15708)	242972 (12495)	246751 (8260)	269405 (12337)	287693 (12379)	9.73E-03	-	-
Cer	Cer(d36:2)	12870 (1074)	12767 (953)	12429 (1136)	12766 (593)	12086 (797)	9936 (465)	1.58E-02	-	-
Cer	Cer(d36:1)	81370 (6258)	85713 (5855)	84296 (6320)	81688 (2742)	80430 (3821)	75494 (4088)	4.59E-02	-	-
Cer	Cer(d44:1)	19464 (817)	20023 (794)	20014 (889)	21264 (732)	22414 (1085)	21310 (509)	NS	NS (0.068)	NS
Cer	Cer(d38:1)	39347 (2231)	43181 (2692)	43859 (3194)	42654 (1446)	48283 (4406)	48237 (1961)	NS	NS	1.97E-02
FFA	FFA 18:2	8997 (891)	9093 (1113)	12767 (674)	8568 (1126)	55878 (7531)	99814 (10837)	6.52E-23	-	-
FFA	FFA 20:0	42010 (1521)	41579 (1825)	35790 (1272)	41060 (1406)	48658 (1461)	52522 (1042)	2.08E-09	-	-
FFA	FFA 18:3	282 (52)	327 (79)	384 (59)	262 (42)	3911 (625)	5575 (984)	3.57E-09	-	-
FFA	FFA 22:0	35633 (1092)	36652 (861)	34575 (903)	35406 (1320)	47067 (1625)	46444 (1720)	1.10E-05	-	-
FFA	FFA 20:3	1058 (93)	1069 (127)	1701 (154)	1047 (185)	1747 (158)	3210 (281)	1.84E-05	-	-
FFA	FFA 18:0	862541 (26597)	866405 (19117)	784450 (22876)	837051 (30993)	833412 (34387)	917003 (27261)	4.83E-04	-	-
FFA	FFA 24:0	74480 (965)	77697 (2129)	74571 (1168)	76472 (1377)	83632 (1970)	87743 (2012)	6.52E-03	-	-
FFA	FFA 16:0	339254 (26711)	298913 (15637)	297798 (16436)	312849 (21798)	332462 (29781)	382341 (20703)	NS (0.051)	NS	NS
FFA	FFA 24:1	6929 (266)	6979 (263)	6768 (352)	6799 (246)	8324 (370)	7642 (259)	NS (0.091)	1.22E-02	NS (0.071)
FFA	FFA 20:4	12616 (1502)	12492 (1767)	20536 (1970)	12907 (2239)	12900 (1332)	24956 (3225)	NS	NS	6.49E-10
FFA	FFA 12:0	3085 (278)	3050 (270)	2772 (328)	2754 (308)	2436 (195)	2749 (228)	NS	NS	NS
FFA	FFA 22:5	1913 (225)	2101 (208)	3048 (251)	2226 (354)	2285 (211)	3635 (342)	NS	NS	4.58E-09
FFA	FFA 22:6	4190 (523)	4118 (508)	6621 (599)	4490 (806)	4007 (410)	7465 (1007)	NS	NS	1.11E-08
FFA	FFA 20:5	1930 (317)	1881 (340)	3080 (398)	1907 (410)	1649 (172)	2616 (436)	NS	NS	1.72E-04
FFA	FFA 14:0	10890 (720)	11217 (844)	11429 (1268)	11839 (973)	13178 (1586)	12279 (855)	NS	NS	NS
FFA	FFA 18:1	149636 (9441)	146819 (17115)	206680 (13195)	144428 (12793)	149421 (15085)	214811 (15219)	NS	NS	1.28E-08
FFA	FFA 20:1	6084 (599)	7541 (912)	8283 (708)	7732 (656)	8912 (733)	9227 (645)	NS	NS	4.45E-02
HexCer	HexCer(d40:1)	12938 (778)	13987 (528)	14002 (676)	12717 (642)	13046 (529)	14258 (927)	NS	NS	NS (0.068)
HexCer	HexCer(d42:2)	29429 (1343)	30501 (1583)	30791 (1932)	31560 (2203)	32678 (2142)	34110 (2436)	NS	NS	9.53E-03
LPC	LPC 24:0	1070 (89)	1161 (112)	1050 (87)	1000 (91)	2983 (190)	5074 (288)	2.16E-35	-	-
LPC	LPC 18:0	36908 (1303)	35238 (1151)	35244 (1563)	35991 (2007)	80348 (4197)	95472 (6966)	1.12E-24	-	-
LPC	LPC 16:0	42812 (1505)	41792 (2707)	41540 (3051)	41156 (2798)	99131 (5459)	114850 (7822)	9.22E-23	-	-
LPC	LPC 20:0	1198 (99)	1202 (88)	1404 (68)	1361 (196)	4106 (250)	4912 (345)	1.39E-22	-	-
LPC	LPC 18:2	214 (27)	214 (38)	214 (44)	233 (32)	17868 (2278)	18758 (2623)	7.19E-17	-	-
LPC	LPC 17:0	604 (37)	524 (56)	602 (53)	568 (33)	1226 (79)	1414 (98)	9.39E-14	-	-
LPC	LPC 20:1	419 (38)	472 (62)	342 (34)	408 (42)	1415 (107)	1115 (114)	2.38E-13	-	-
LPC	LPC 20:4	729 (127)	615 (148)	389 (79)	490 (51)	1877 (201)	1542 (247)	8.77E-10	-	-
LPC	LPC 18:1	11396 (771)	10551 (1341)	8400 (1018)	10130 (793)	19273 (1532)	20252 (2354)	3.16E-07	-	-
LPC	LPC 14:0	1895 (117)	1877 (226)	1627 (169)	2093 (205)	970 (79)	648 (57)	3.71E-06	-	-
LPC	LPC 15:0	2043 (100)	2141 (173)	2169 (120)	2109 (99)	2184 (144)	1963 (128)	NS	NS	NS
LPE	LPE 18:0	5289 (224)	4964 (279)	5443 (287)	5208 (352)	9917 (457)	12084 (772)	5.96E-22	-	-
LPE	LPE 16:0	2232 (140)	2276 (166)	2483 (254)	2349 (183)	6263 (460)	8099 (687)	5.24E-17	-	-
LPE	LPE 18:1	17369 (1487)	16638 (1879)	18227 (1308)	17757 (2200)	18723 (986)	22191 (1445)	NS	NS	NS
LPI	LPI 18:0	227 (27)	282 (31)	306 (45)	252 (35)	330 (35)	677 (117)	1.64E-03	-	-
LPI	LPI 20:4	500 (107)	450 (128)	940 (153)	431 (145)	518 (127)	1642 (211)	1.21E-02	-	-
LPI	LPI 18:1	1013 (206)	1215 (384)	2736 (539)	1007 (296)	710 (210)	1607 (285)	NS	NS	1.56E-04

Class	Metabolite	Control			Intralipid			FDR corrected P-values		
		day 0	day 4	day 8	day 0	day 4	day 8	Interaction	Group	Time
LPS	LPS 18:0	1562 (233)	1523 (251)	2271 (290)	1551 (302)	1366 (143)	3070 (396)	NS	NS	2.16E-07
LPS	LPS 20:4	11182 (1106)	11080 (1027)	15629 (1162)	12030 (1524)	8717 (758)	13248 (1418)	NS	NS	2.54E-06
LPS	LPS 18:1	22537 (2377)	23309 (2916)	34582 (3153)	22381 (2511)	18462 (2128)	29768 (2702)	NS	NS	5.64E-06
PC	PC(18:1_18:2)	1040 (174)	1118 (181)	996 (152)	1096 (161)	13590 (1719)	11352 (1472)	9.72E-14	-	-
PC	PC(16:0_18:2)	11104 (545)	11309 (473)	9704 (409)	11022 (934)	30033 (2843)	28766 (3663)	4.40E-11	-	-
PC	PC(p-32:1) / PC(o-32:2)	179220 (7474)	175227 (7712)	176790 (7890)	185495 (6313)	163010 (6169)	147612 (6598)	5.20E-08	-	-
PC	PC(20:0_18:2)	5990 (265)	6234 (172)	5333 (135)	6013 (333)	5771 (281)	7145 (270)	3.06E-05	-	-
PC	PC(18:0_22:6)	3804 (269)	3903 (209)	4130 (277)	4126 (260)	3370 (194)	3227 (127)	1.21E-02	-	-
PC	PC 42:10	11445 (644)	11950 (515)	12113 (460)	12011 (574)	11180 (473)	9923 (429)	1.61E-02	-	-
PC	PC(18:0_18:1)	6328 (600)	7751 (502)	6357 (521)	6309 (871)	4782 (409)	5104 (625)	1.61E-02	-	-
PC	PC(18:2_22:5)	15869 (1248)	17845 (1071)	18040 (1024)	17215 (913)	15060 (579)	14918 (875)	1.75E-02	-	-
PC	PC(p-38:4) / PC(o-38:5)	3379 (352)	4410 (421)	2766 (273)	3073 (418)	2579 (428)	1354 (190)	3.32E-02	-	-
PC	PC(18:0_22:5)	19101 (337)	19305 (377)	19497 (397)	19053 (566)	17689 (376)	17458 (398)	NS (0.05)	6.37E-03	NS
PC	PC(16:1_18:2)	4746 (585)	4595 (572)	4620 (591)	5740 (475)	8236 (513)	7381 (620)	NS (0.076)	1.51E-05	NS
PC	PC(20:4_20:4)	27898 (1204)	29427 (2483)	28076 (1535)	29400 (1518)	26550 (1191)	27084 (1282)	NS	NS	NS
PC	PC(18:1_18:1)	16232 (1131)	16642 (1461)	13056 (581)	15362 (1752)	16374 (1627)	16398 (1996)	NS	NS	NS
PC	PC(p-38:5) / PC(o-38:6)	3076 (218)	3534 (165)	3175 (213)	2988 (181)	2811 (180)	2325 (249)	NS	3.00E-03	NS
PC	PC(18:0_20:4)	5083 (408)	4412 (370)	4600 (324)	4149 (225)	4202 (332)	4564 (310)	NS	NS	NS
PC	PC(p-34:0) / PC(o-34:1)	23295 (2352)	25580 (2804)	14134 (1608)	19823 (3870)	14681 (2850)	10646 (2456)	NS	NS (0.091)	2.36E-04
PC	PC(14:0_18:2)	117560 (2653)	117554 (3403)	120314 (3695)	123605 (2248)	117766 (3083)	117752 (2131)	NS	NS	NS
PC	PC(18:1_20:3)	5766 (594)	5837 (407)	5745 (548)	5097 (274)	5040 (439)	6069 (299)	NS	NS	NS
PC	PC(p-36:1) / PC(o-36:2)	8353 (601)	9804 (1058)	6459 (718)	7816 (977)	6919 (1208)	4561 (888)	NS	NS	4.35E-04
PC	PC(16:0_18:1)	28064 (2251)	29018 (3250)	19841 (1749)	27536 (5179)	22234 (2742)	18564 (3119)	NS	NS	5.10E-03
PC	PC(16:0_20:5)	4569 (344)	4549 (456)	3968 (234)	4316 (387)	3716 (209)	3418 (318)	NS	NS	1.20E-02
PC	PC(16:1_22:6)	132476 (6296)	136621 (8345)	141479 (8599)	147440 (10543)	155586 (10520)	169195 (8832)	NS	NS	NS (0.058)
PC	PC(16:0_16:0)	7278 (632)	7929 (901)	7113 (761)	7124 (985)	5926 (499)	5921 (1776)	NS	NS	NS
PC	PC(p-36:5) / PC(o-36:6)	14662 (852)	14674 (672)	15183 (826)	13772 (627)	13492 (638)	13402 (799)	NS	NS	NS
PE	PE 36:3	190070 (10729)	187971 (8622)	203485 (10857)	195409 (8245)	351425 (13268)	498003 (17322)	5.98E-86	-	-
PE	PE(16:1_22:5)	101266 (6904)	114550 (4549)	106887 (7299)	121097 (4145)	216241 (7352)	306250 (11622)	2.20E-38	-	-
PE	PE 38:5	570294 (18860)	557291 (19652)	538766 (18584)	604304 (13333)	531565 (13241)	451041 (12866)	7.14E-10	-	-
PE	PE 32:1	40179 (1187)	44332 (992)	43652 (1590)	46097 (2081)	43269 (1307)	38499 (1061)	1.43E-06	-	-
PE	PE(16:0_18:2)	135773 (5140)	135088 (4319)	132786 (5501)	144867 (5374)	156504 (6213)	182598 (7325)	1.46E-06	-	-
PE	PE 32:0	2008 (170)	2163 (198)	2032 (147)	2348 (247)	2443 (161)	3261 (226)	1.71E-03	-	-
PE	PE 40:6	105516 (3827)	103550 (3698)	104018 (4037)	108767 (4002)	100977 (3679)	92394 (2686)	3.48E-03	-	-
PE	PE O-40:5/P-40:4/39:5	109244 (4504)	102067 (5509)	95212 (5377)	100240 (6295)	101134 (4899)	109336 (5372)	1.17E-02	-	-
PE	PE(18:0_18:1)	262374 (9145)	265282 (10757)	259026 (11049)	281794 (9496)	261960 (10486)	241360 (6644)	1.58E-02	-	-
PE	PE(16:1_20:3)	71279 (2512)	71433 (1739)	71082 (1523)	75974 (2593)	72502 (2388)	66861 (1747)	2.25E-02	-	-
PE	PE 36:5	15318 (1094)	14921 (1123)	15365 (1162)	16141 (1746)	14250 (1022)	12850 (848)	NS (0.056)	NS	NS (0.057)
PE	PE 34:4	4140 (162)	4477 (327)	4737 (209)	4644 (316)	4914 (296)	4287 (166)	NS (0.085)	NS	NS
PE	PE O-34:3/P-34:2	16082 (523)	17091 (1046)	16674 (925)	17278 (1424)	15900 (753)	14843 (762)	NS	NS	NS
PE	PE 36:6	5134 (262)	6338 (323)	6306 (220)	6506 (587)	6246 (358)	7610 (522)	NS	NS (0.075)	1.44E-02
PE	PE 38:3	29914 (874)	32668 (1976)	30876 (2000)	34405 (1408)	32758 (1203)	31724 (1240)	NS	NS	NS
PE	PE O-36:4/P-36:3	401161 (17665)	399651 (17269)	386595 (10162)	387459 (22422)	354120 (13084)	343801 (18582)	NS	NS	1.06E-02
PE	PE O-38:5/P-38:4	21189 (1739)	21408 (1397)	19084 (1148)	22015 (3602)	17304 (1595)	15420 (1759)	NS	NS	2.13E-02
PE	PE 38:2	40934 (1715)	41757 (1614)	40292 (1804)	42776 (1930)	41000 (1422)	39598 (913)	NS	NS	NS

Class	Metabolite	Control day 0	Control day 4	Control day 8	Intralipid day 0	Intralipid day 4	Intralipid day 8	FDR corrected P-values Interaction	FDR corrected P-values Group	FDR corrected P-values Time
PE	PE 38:1	2360 (138)	2635 (249)	2502 (145)	2595 (174)	2392 (121)	2635 (120)	NS	NS	NS
PE	PE(18:0_18:2)	1744220 (54029)	1694682 (59167)	1601441 (57954)	1805700 (51293)	1691270 (54875)	1561123 (41827)	NS	NS	3.17E-09
PE	PE O-36:3/P-36:2/35:3	144411 (7163)	150433 (8370)	147584 (8207)	155361 (11691)	147992 (8548)	150376 (10403)	NS	NS	NS
PE	PE 40:2	5014 (296)	5448 (371)	4938 (343)	4856 (307)	4590 (217)	4605 (241)	NS	NS	NS
PE	PE 34:3	11596 (418)	11635 (544)	11395 (791)	12309 (824)	12773 (521)	11030 (519)	NS	NS	NS
PE	PE O-36:6/P-36:5	14981 (1077)	15390 (892)	14830 (1150)	15020 (1477)	14451 (720)	12951 (751)	NS	NS	NS
PE	PE 34:1	650910 (16974)	665422 (19238)	638240 (23557)	697411 (22143)	672772 (24247)	646847 (13524)	NS	NS	NS
PE	PE 42:9	11849 (482)	12164 (389)	11207 (585)	12227 (627)	11840 (528)	12189 (603)	NS	NS	NS
PE	PE O-38:7/P-38:6	29567 (1496)	30394 (2000)	29634 (1559)	30308 (2877)	30182 (2088)	31244 (2500)	NS	NS	NS
PE	PE O-38:4/P-38:3/37:4	264871 (11651)	253705 (6930)	240030 (6759)	264730 (8918)	246588 (7446)	245353 (6790)	NS	NS	1.10E-03
PE	PE O-34:2/P-34:1	242912 (12442)	244423 (14450)	240102 (14105)	244324 (17302)	239323 (13894)	242976 (16542)	NS	NS	NS
PE	PE 40:5	14954 (655)	14914 (513)	13875 (471)	15484 (897)	15566 (922)	13949 (710)	NS	NS	9.47E-04
PE	PE O-40:6/P-40:5/39:6	103151 (6377)	99134 (7819)	96107 (6986)	95176 (7250)	89552 (6327)	89460 (6626)	NS	NS	NS
PE	PE(18:0_20:4)	87452 (5855)	84848 (3621)	81544 (2744)	92603 (5415)	90883 (4233)	89339 (3365)	NS	NS	NS
PE	PE O-38:6/P-38:5	252937 (14104)	246782 (19246)	238242 (17900)	244102 (23718)	238929 (19183)	225781 (17492)	NS	NS	NS
PE	PE O-36:5/P-36:4	23023 (1848)	23036 (1313)	21450 (1673)	21397 (1321)	21107 (1657)	19389 (1589)	NS	NS	NS
PG	PG 34:1	14251 (799)	14652 (927)	14379 (704)	15003 (1401)	14706 (1209)	15566 (1161)	NS	NS	NS
PG	PG 36:2	4608 (318)	4529 (295)	4781 (230)	5157 (524)	4972 (370)	4931 (397)	NS	NS	NS
PG	PG 36:1	3723 (331)	3659 (460)	3233 (280)	3911 (600)	3666 (354)	3241 (483)	NS	NS	NS (0.091)
PG	PG 36:0	59206 (3865)	56710 (4792)	52768 (3934)	53314 (4819)	49731 (4140)	45755 (4259)	NS	NS	3.67E-03
PI	PI 36:3	27696 (1363)	30226 (1321)	33857 (3267)	29220 (1265)	59746 (2386)	89828 (2134)	2.45E-41	-	-
PI	PI 34:2	64277 (1779)	66831 (2057)	70118 (3117)	67288 (2184)	84417 (2363)	109859 (2583)	1.09E-21	-	-
PI	PI 36:4	40690 (2074)	39057 (1267)	38311 (1361)	40929 (2201)	56174 (3044)	64229 (4435)	2.26E-12	-	-
PI	PI 38:5	63395 (3382)	64075 (2867)	65864 (3308)	63708 (2328)	82415 (3043)	85977 (3337)	2.92E-08	-	-
PI	PI 30:0	7542 (403)	8894 (686)	9277 (835)	7843 (632)	7106 (539)	5998 (563)	3.25E-04	-	-
PI	PI 32:1	48155 (1683)	49369 (1862)	50802 (2445)	48709 (2080)	46128 (1808)	41089 (2285)	1.53E-03	-	-
PI	PI 40:6	8707 (607)	9340 (646)	9437 (584)	8692 (424)	7783 (329)	6962 (427)	2.15E-03	-	-
PI	PI 40:4	12860 (666)	13893 (599)	13975 (704)	13647 (619)	12430 (502)	11187 (476)	1.14E-02	-	-
PI	PI 36:1	513434 (26799)	557261 (27437)	569059 (29854)	542502 (30071)	518474 (25741)	492065 (14673)	2.46E-02	-	-
PI	PI 38:6	19060 (1065)	20047 (1076)	21151 (1454)	19023 (1086)	17993 (942)	16750 (1142)	4.71E-02	-	-
PI	PI 36:5	6119 (403)	5969 (445)	6772 (724)	5756 (394)	7497 (879)	8093 (770)	NS (0.052)	NS	1.11E-03
PI	PI 38:3	68915 (5510)	70907 (7297)	68085 (4692)	68917 (5001)	61163 (4712)	56209 (3757)	NS	NS	NS
PI	PI 34:1	274368 (8676)	286048 (7332)	279192 (9820)	268381 (7873)	262707 (7487)	253472 (8868)	NS	NS	NS
PI	PI 40:5	27688 (1437)	27801 (886)	27316 (1129)	28197 (1460)	26421 (1141)	24781 (877)	NS	NS	NS
PI	PI 32:0	46194 (2031)	49221 (2557)	47251 (2585)	44504 (2391)	45769 (2311)	42804 (2468)	NS	NS	NS
PI	PI 36:2	334102 (15233)	356158 (18063)	348937 (17261)	357141 (22639)	358866 (22555)	378203 (14342)	NS	NS	NS
PI	PI 38:4	87980 (5609)	91313 (3452)	88947 (3416)	88579 (2772)	96351 (3893)	96741 (4659)	NS	NS	NS
PI	PI 38:2	31458 (1327)	32605 (1043)	31712 (1167)	33354 (1008)	34658 (1378)	32559 (1607)	NS	NS	NS
PI	PI 34:0	18452 (1374)	19243 (1326)	19369 (1068)	19958 (1255)	20265 (1647)	20756 (1414)	NS	NS	NS
PS	PS 36:3	174679 (5332)	178681 (4679)	195937 (11547)	173165 (7296)	245013 (5831)	316273 (7732)	1.29E-25	-	-
PS	PS 34:2	137610 (4158)	142172 (4262)	140491 (3500)	137975 (5319)	159563 (4340)	197086 (6515)	2.88E-13	-	-
PS	PS 34:3	4393 (474)	4161 (314)	4701 (573)	4809 (607)	7341 (452)	10811 (303)	3.03E-09	-	-
PS	PS 38:5	296321 (10605)	309800 (10583)	303191 (9385)	312361 (12903)	285284 (9655)	250960 (8593)	1.31E-06	-	-
PS	PS 40:7	40777 (2219)	42263 (1958)	39904 (1648)	43440 (3152)	39096 (1903)	33590 (2165)	2.15E-04	-	-
PS	PS 32:1	62942 (1985)	68714 (2337)	65172 (2188)	63286 (2063)	61553 (1304)	55537 (1532)	1.13E-03	-	-

		Control			Intralipid			FDR corrected P-values		
**Class**	**Metabolite**	**day 0**	**day 4**	**day 8**	**day 0**	**day 4**	**day 8**	**Interaction**	**Group**	**Time**
PS	PS 38:4	140031 (5840)	144285 (4890)	145938 (4250)	147372 (4429)	137452 (4948)	125810 (4866)	2.49E-03	-	-
PS	PS 40:2	8805 (383)	9748 (533)	9603 (351)	9121 (230)	8441 (193)	8481 (318)	4.47E-03	-	-
PS	PS 34:1	1303509 (39468)	1349016 (46515)	1297524 (40731)	1305101 (40047)	1187910 (26700)	1108365 (30979)	1.47E-02	-	-
PS	PS 40:3	6152 (343)	7797 (809)	6939 (561)	7134 (435)	6667 (438)	6240 (524)	NS	NS	NS
PS	PS 38:1	19909 (782)	20520 (969)	20692 (1265)	19776 (396)	19071 (443)	18535 (641)	NS	NS	NS
PS	PS 38:2	65559 (1652)	66482 (2223)	64658 (1837)	64291 (1343)	61577 (1182)	59631 (1011)	NS	NS (0.091)	NS
PS	PS 36:2	1315100 (25392)	1339634 (34019)	1319320 (35712)	1338607 (33293)	1303639 (29050)	1342503 (20947)	NS	NS	NS
PS	PS 40:6	22054 (2944)	21361 (3257)	18777 (2488)	18465 (2817)	18188 (2267)	17201 (2189)	NS	NS	NS (0.091)
PS	PS 38:3	64113 (4089)	67388 (1941)	72079 (3189)	69963 (1371)	70240 (1931)	75369 (1821)	NS	NS	6.28E-03
PS	PS 36:1	1219026 (29286)	1240476 (37351)	1180420 (30718)	1249192 (18922)	1253820 (14050)	1181965 (14173)	NS	NS	2.06E-03
PS	PS 38:6	66386 (4420)	67644 (4061)	68308 (3568)	65462 (5057)	65389 (4015)	66165 (3876)	NS	NS	NS
PS	PS 34:0	13003 (621)	13483 (648)	14232 (745)	15174 (1031)	15167 (866)	16408 (865)	NS	NS (0.068)	NS
PS	PS 40:5	31691 (2581)	30567 (3240)	28576 (2411)	29417 (2411)	27574 (1700)	26340 (1444)	NS	NS	NS (0.091)
PS	PS 40:4	14428 (856)	14211 (566)	14696 (961)	14729 (731)	14449 (576)	15162 (424)	NS	NS	NS
PS	PS 30:0	1527 (100)	1610 (160)	1560 (103)	1607 (147)	1712 (78)	1656 (94)	NS	NS	NS
SM	SM(d36:1)	323318 (10291)	334219 (11631)	343739 (10619)	330018 (8511)	217274 (5312)	153404 (4789)	4.24E-75	-	-
SM	SM(d32:2)	3778 (201)	3694 (221)	3611 (190)	3897 (219)	2360 (233)	1613 (204)	8.22E-17	-	-
SM	SM(d36:2)	53496 (1223)	54026 (1862)	54849 (932)	52441 (1760)	39204 (1354)	30833 (1090)	1.02E-16	-	-
SM	SM(d32:1)	93327 (2691)	94877 (4399)	91809 (4670)	101951 (3363)	80520 (4215)	68561 (2469)	9.56E-08	-	-
SM	SM(d38:2)	25806 (923)	26731 (1014)	27467 (1339)	26754 (929)	23347 (791)	20521 (783)	6.75E-07	-	-
SM	SM(d36:0)	14542 (862)	15754 (947)	15260 (1051)	15156 (953)	13077 (741)	11906 (913)	2.06E-05	-	-
SM	SM(d36:3)	14522 (872)	15787 (961)	15243 (1073)	15160 (972)	13102 (746)	11998 (927)	4.04E-05	-	-
SM	SM(d34:2)	151958 (2856)	152227 (4284)	148948 (5314)	152882 (3792)	140937 (4559)	134793 (4832)	NS (0.06)	NS	5.10E-03
SM	SM(d41:1)	46051 (778)	47661 (1365)	48300 (1521)	46469 (928)	48140 (716)	51746 (820)	NS	NS	2.35E-06
SM	SM(d43:2)	107286 (1779)	110631 (2286)	113420 (3302)	107233 (942)	106235 (2160)	105540 (1847)	NS	NS	NS
SM	SM(d40:2)	103777 (4466)	110082 (5058)	114159 (5678)	110852 (3557)	114122 (3103)	109279 (3497)	NS	NS	NS
SM	SM(d34:0)	88605 (4092)	92426 (4230)	90782 (3696)	91382 (3427)	89372 (3068)	89184 (3753)	NS	NS	NS
SM	SM(d42:2)	2182408 (37239)	2215094 (47154)	2285833 (48205)	2235006 (62180)	2209669 (33854)	2207659 (31313)	NS	NS	NS
SM	SM(d34:1)	1974569 (33946)	2039508 (35851)	1996375 (45708)	2033224 (33513)	2013696 (31407)	2028802 (36959)	NS	NS	NS
SM	SM(d33:1)	50820 (2010)	50751 (3008)	51575 (1968)	51466 (1595)	47521 (1616)	48586 (2137)	NS	NS	NS
SM	SM(d44:2)	319863 (7183)	324055 (6939)	322054 (7097)	326248 (9858)	321662 (7177)	316939 (9195)	NS	NS	NS
SM	SM(d40:0)	30264 (912)	30171 (1180)	30670 (1437)	30653 (863)	32890 (852)	32031 (672)	NS	NS	NS
SM	SM(d41:2)A	197767 (5254)	203023 (5413)	203854 (3399)	202306 (3483)	198945 (3183)	200266 (4245)	NS	NS	NS
SM	SM(d43:1)	13217 (509)	13346 (496)	13895 (473)	13220 (515)	14126 (331)	14092 (523)	NS	NS	NS
SM	SM(d42:1)	505356 (12759)	513220 (14018)	522255 (16606)	509222 (14751)	520453 (15681)	518607 (13695)	NS	NS	NS
SM	SM(d41:2)B	224798 (4288)	225781 (4375)	216932 (6029)	226069 (4448)	224287 (5847)	221773 (5836)	NS	NS	NS
SM	SM(d38:1)	34740 (925)	34350 (1176)	33551 (1323)	34507 (752)	35176 (576)	33776 (837)	NS	NS	NS
SM	SM(d40:1)	250976 (6326)	256803 (7901)	260677 (8089)	258086 (4288)	262102 (4521)	266568 (5519)	NS	NS	NS

Relative concentrations of lipids within fetal RBCs were determined by LC-MS. Data are mean (SEM) of normalized peak areas. P-values are derived from linear mixed models with infusion group and time as main effects and an interaction term of group by time. Intra-individual variation was included as random effect. P-values for each term were adjusted for multiple comparisons by the Benjamini and Hochberg false discovery rate correction. Ceramide (Cer). False discovery rate (FDR). Free fatty acid (FFA). Hexosylceramide (HexCer). Lysophosphatidylcholine (LPC). Lysophosphatidylethanolamine (LPE). Lysophosphatidylinositol (LPI). Not significant (NS). Phosphatidylcholine (PC). Phosphatidylethanolamine (PE). Phosphatidylglycerol (PG). Phosphatidylinositol (PI). Phosphatidylserine (PS). Sphingomyelin (SM).
